# Comprehensive Analysis of a Yeast Lipase Family in the *Yarrowia* Clade

**DOI:** 10.1371/journal.pone.0143096

**Published:** 2015-11-18

**Authors:** Muchalin Meunchan, Stéphanie Michely, Hugo Devillers, Jean-Marc Nicaud, Alain Marty, Cécile Neuvéglise

**Affiliations:** 1 Université de Toulouse, INSA, UPS, INP, LISBP, 135 Avenue de Rangueil, F-31077, Toulouse, France; 2 INRA, UMR792 Ingénierie des Systèmes Biologiques et des Procédés, F-31400, Toulouse, France; 3 CNRS, UMR5504, F-31400, Toulouse, France; 4 Department of Biochemistry, Faculty of Science, Khon Kaen University, 123 Mittapap Road, Khon Kaen, 40002, Thailand; 5 INRA, UMR 1319 Micalis, F-78352, Jouy-en-Josas, France; 6 AgroParisTech, UMR Micalis, F-78352, Jouy-en-Josas, France; University of Leicester, UNITED KINGDOM

## Abstract

Lipases are currently the subject of intensive studies due to their large range of industrial applications. The Lip2p lipase from the yeast *Yarrowia lipolytica* (Yl*LIP2*) was recently shown to be a good candidate for different biotechnological applications. Using a combination of comparative genomics approaches based on sequence similarity, synteny conservation, and phylogeny, we constructed the evolutionary scenario of the lipase family for six species of the *Yarrowia* clade. RNA-seq based transcriptome analysis revealed the primary role of LIP2 homologues in the assimilation of different substrates. Once identified, these Yl*LIP2* homologues were expressed in *Y*. *lipolytica*. The lipase Lip2a from *Candida phangngensis* was shown to naturally present better activity and enantioselectivity than YlLip2. Enantioselectivity was further improved by site-directed mutagenesis targeted to the substrate binding site. The mono-substituted variant V232S displayed enantioselectivity greater than 200 and a 2.5 fold increase in velocity. A double-substituted variant 97A-V232F presented reversed enantioselectivity, with a total preference for the *R*-enantiomer.

## Introduction

Lipases are serine hydrolases defined as triacylglycerol acylhydrolases (E.C. 3.1.1.3). They are ubiquitous enzymes of high physiological significance and their industrial potential is on the increase. Their physiological role is to catalyse the hydrolysis of the ester bond of tri-, di- and monoglycerides of long-chain fatty acids into fatty acids and glycerol [1]. Lipases are widely used as additives in industrial laundry and household detergents [2], in the food industry for, for instance, the development of flavours, the production of structured lipids or the production of oil enriched in polyunsaturated acids, paper manufacture, degradation of fatty waste [3], and in the synthesis of fine chemicals, cosmetics and pharmaceuticals.

Fungi are known to secrete lipases to assimilate lipid substrates from their natural environment. Being ubiquitous, lipases have developed substrate specificities and stability under a range of chemical and physical conditions, which increases their interest for industrial applications. In addition, some fungal lipases are extracellular, thus reducing the cost of production and making this source preferable over bacteria. Among fungal lipases [4], those of ascomycetous yeasts from various species such as *Candida rugosa* [5], *Yarrowia* (*Candida*) *deformans* [6], *Candida albicans* [7], *Candida viswanathii* [8], or *Yarrowia lipolytica* [9] have been widely studied, as well as species from Basidiomycota such as *Pseudozyma* (*Candida*) *antarctica* [10] or from Mucoromycotina with *Rhizomucor* (*Mucor*) *miehei* as an example. The latter species was the first used for polyunsaturated fatty acids (PUFAs) concentrate production, including docosahexaenoic acid (DHA) [11]. The lipases in most of these species belong to multigenic families. Up to 16 members have been reported in *Y*. *lipolytica*, but only the extracellular YlLip2 lipase has been extensively studied [12], and was reported to be an efficient enzyme in a number of applications [13]. For instance, it was demonstrated to be the most effective lipase for the purification of *cis*-4,7,10,13,16,19-docosahexaenoic acid (DHA) by hydrolysis of a mixture of ethyl esters from tuna oil [14] and to be an efficient stereo-selective enzyme for the resolution of 2-halogeno-arylacetic acid esters, an important class of chemical intermediates in the pharmaceutical industry [15]. Additionally *Y*. *lipolytica* has been shown to have multiple industrial applications in other fields of biotechnology such as waste treatment, traditional food making, citric acid production (reviewed in [9, 16]), as well as production of chemicals, fuels and specific lipids such as PUFAs containing omega-3 and omega-6 fatty acids for human and animal feed [17].

Studies have been conducted to improve YlLip2 properties, e.g. lipase activity, enantioselectivity, and thermostability. The strategies used were mainly based on site-directed mutagenesis targeted to the substrate binding site [18] or on random mutagenesis of the whole gene sequence [19]. For instance, the mono-substituted variant with the valine 232 changed into serine in YlLip2, represents a tremendous increase in enantioselective activity compared to the parental enzyme for the resolution of 2-bromo-phenylacetic acid ethyl ester (58-fold) and 2-bromo-o-tolylacetic acid ethyl ester (16-fold) [18]. This type of experimental approach requires the screening of a large set of mutants and is thus laborious and, in addition, the outcome is uncertain.

In this study, we investigated the natural biodiversity of lipases in species closely related to *Y*. *lipolytica*. The approach used takes advantage of the decreasing cost of genome sequencing, thereby enabling a huge number of closely related organisms to be sequenced. *Y*. *lipolytica* is an interesting reservoir of lipases of which only few members have been exploited to date. These paralogue lipases, which derived from gene duplications, are already too divergent to deduce relationships between activities and amino-acid sequences. An alternative approach is to identify more conserved lipases derived from a common ancestor, which could have acquired improved specificities. Combining comparative genomics and phenotypic characterisation may provide clues to interesting selection of lipases with specificities of high interest for industry. From this perspective, we investigated the genomes of six members of the *Yarrowia* clade and detected 61 lipase genes belonging to different LIP families. The study of the evolution of the lipases made it possible to identify the orthologous relationships between homologues and to retrace their evolutionary history. With 11 members closely related to YlLip2, the Lip2 family is the largest and the most dynamic one. We consequently investigated this family and tried to infer the specificities of each member by over-expressing them in *Y*. *lipolytica* under the control of the strong constitutive TEF promoter and to identify the best lipase for resolution of 2-halogeno-arylacetic acid esters.

## Materials and Methods

### Strains

The wild-type prototroph strains of the *Yarrowia* clade investigated in this study were the following (abbreviations of strains are within brackets): *Y*. *lipolytica* W29 = CBS 7504 (YALI), *Y*. *yakushimensis* CBS 10253 (YAYA), *Y*. *deformans* CBS 2071 (YADE), *Y*. *galli* CBS 9722 (YAGA), *Y*. *oslonensis* CBS 10146 (YAOS), *Y*. *hollandica* CBS 4855 (YAHO), *Y*. *phangngensis* CBS 10407 (YAPH), and *Y*. *alimentaria* CBS 10151 (YAAL). In addition, *Candida hispaniensis* CBS 9996 (CAHI) was used as the nearest out-group species for the *Yarrowia* clade (Michely et al., in prep). Strains used for lipase expression in *Y*. *lipolytica* are listed in Table 1.

**Table 1:** Strains used for evolutionary analysis.

Species	Strain number	Reference
*Yarrowia lipolytica*	CBS 7504 = W29	[41]
*Candida galli*	CBS 9722	[53]
*Yarrowia yakushimensis*	CBS 10253	[54]
*Candida phangngensis*	CBS 10407	[55]
*Candida alimentaria*	CBS 10151	[56]
*Candida hispaniensis*	CBS 9996	[57]

CBS: Centraalbureau voor Schimmelcultures, Utrecht, The Netherlands

### Identification of lipases in species of the *Yarrowia* clade

The genomes of YAGA, YAYA, YAPH, YAAL, and CAHI were sequenced and annotated at INRA Thiverval-Grignon. Genome annotations were performed by a combination of *in silico* annotation transfer tool based on Amadea BioPack (ISoft, France) and manual curation based on RNA-Seq data. Genes encoding lipases (LIP genes) were found by homology using the LIP genes of YALI [20]. Orthologues in each species were identified based on a two-step reciprocal approach using BLASTP. In the first step, YALI LIP genes were used as queries for BLASTP on the 5 other species, with a cutoff E-value of 1.e^-10^ [21]. Then a reciprocal BLASTP search considering only the best hit allowed genes corresponding to other YALI genes to be discarded. The sequence of all LIP genes have been deposited at the European Nucleotide Archive ENA-EMBL under accession numbers LM652719- LM652763. Their protein sequences are provided in S1 File.

### RNA-Seq analysis

YALI, YAGA and YAPH were used for transcriptome analysis in three different media, i.e. glucose (GL), oleic acid (OA) and tributyrin (TB). The strains were cultured at 28°C on YPD medium (1% (wt/vol) yeast extract, 1% (wt/vol) peptone, 1% (wt/vol) glucose) for precultures. Then, minimal medium base (MMB) was used, containing 0.17% (wt/vol) yeast nitrogen base without amino acids and ammonium sulfate (Difco, Paris, France), 0.5% (wt/vol) NH_4_Cl, 50 mM phosphate buffer pH 6.8 and 0.15% (wt/vol). The three carbon sources (GL, AO, TB) were added at a final concentration of 1%. Both lipid substrates were previously emulsified by sonication of a 20% mixture in the presence of 0.625% Tween 40. Cells grown in the presence of OA or TB were washed twice with 0.5% bovine serum albumin and then once with 0.9% NaCl before OD_600_ determination. Cells were harvested in the exponential phase. Two replicates were performed for each species/media pair. Total RNA was prepared using the Qiagen RNeasy kit (Qiagen, Courtaboeuf, France). mRNA was purified by selection of poly(A)+ transcripts, which were then sequenced by the Illumina Solexa technology with a HiSeq 2000 sequencing system with chemistry v3.0. Twelve to 29 million single-end reads of 100 bp were generated per sample. Sequences were cleaned and trimmed using Trimmomatic version 0.20 (http://www.usadellab.org/cms/index.php?page=trimmomatic) with arguments -threads 20 and -phred33. Only reads with a minimum length of 40 bp, which corresponded to about 98% of the raw data, were further analysed. Tophat2 v2.0.10 with Bowtie2 v2.1.0 were used to map the reads on the nuclear genome of the three strains [22]. The options used were the following: —micro-exon-search —min-intron-length 30 —min-coverage-intron 30 —min-segment-intron 30 —max-intron-length 4000 —max-multihits 1 with the transcriptome being provided. Reads were counted using a custom BioPerl script based on samtools utilities v0.1.18 [23]. In a preliminary step, low expression features were filtered as they generally cause serious biases in differential expression studies. To this end, we followed the procedure described in the egdeR user guide [24]. Thus, only features with at least one count per million (cpm) in at least two conditions/replicates were kept. Differential expression was performed with DEseq2 v1.2.8 [25]. The default normalization proposed by the DEseq2 package was used and the “parametric” estimation of data dispersion was chosen. All the pairwise comparisons of the three different conditions for the three species were investigated. The resulting p-values were corrected using the Benjamini-Hochberg procedure [26]. Assuming that the variability between replicates was lower in YALI than in YAGA and YAPH (data not shown), the adjusted P-value cut-off for the differential expression was fixed at 1.e-3 in YALI and 5.e-3 in YAGA and YAPH. Fastq files of the RNA-Seq reads have been deposited at the European Nucleotide Archive (ENA-EMBL) under the project number PRJEB6632 (http://www.ebi.ac.uk/ena/data/view/ERP006181).

### Reconstruction of the evolutionary scenario

To reconstruct the evolutionary scenario of the lipase family in the *Yarrowia* clade using CAHI as out-group, the species tree and the LIP tree were reconciled with synteny data. A parsimonious approach was used to minimise the number of duplications and loss of LIP genes. For the phylogeny of the LIP genes, protein sequences were aligned with MAFFT [27]. The pairwise comparisons of both amino acid identity and similarity were calculated using aligncopypair (EMBOSS) [28].

The phylogenetic tree deduced from the LIP protein alignment was constructed with PhyML v3.0 with a JTT substitution model corrected for heterogeneity among sites by a Γ-law distribution using 4 different categories of evolution rates [29]. The proportion of invariable sites and the α-parameter of the Γ-law distribution were optimised according to the data. A bootstrap value was calculated with 100 replicates. The species tree was deduced from the alignment of 912 single copy protein-coding genes (398959 residues). Individual gene alignments for the 912 orthologues was performed with MUSCLE [20] edited using Gblocks [30], concatenated, and the phylogenetic tree was estimated by maximum likelihood using PhyML v3.0 assuming a JTT substitution model with Γ distributed rate variation and a proportion of invariant sites estimated from the data.

Synteny conservation between LIP genes was determined by pair-wise comparison between 50 kbp regions upstream and downstream from each LIP gene, using Promer and mummerplot from MUMmer v3.23 [31].

### Lip2 protein sequence analysis

Lip2 proteins were aligned with Multalin [32]. Pairwise identity and similarity between Lip2 proteins were deduced from this alignment using aligncopypair (EMBOSS) [28]. The secondary structure of the Lip2 proteins was predicted with PSIPRED v3.3 [33]. β-sheets were numbered according to the nomenclature of [34] and the α/β hydrolase fold according to [35].

### Selection and dN/dS ratio

Pairwise nucleotide alignment was deduced from MAFFT protein alignment using tranalign in the EMBOSS package [28]. The dN/dS ratios were calculated from this alignment using Codeml model from PAML4 package version 4.4b with a run mode equal to -2 [36]. Saturated dS values were removed from the analysis, which notably excluded all pairwise comparisons involving CAHI. Box plots were constructed with R [37].

### Chemical reagents

Yeast extract, tryptone, and peptone were purchased from Difco (Paris, France). All reagents used for enzymatic reactions were of commercial quality and were purchased from Sigma-Aldrich (St. Louis, MO, US). *n-*Decane was dried over molecular sieve (3 Å) before use. The preparation procedure for (±) 2-bromo-phenylacetic acid octyl ester is described in a previous paper [38].

### Construction of mutants for production of recombinant enzymes

Plasmid JMP62-*URA3*ex-pTEF was used for the production of recombinant enzymes, wild-type and variants [39]. This plasmid carries the wild-type/mutated *LIP2* genes under the control of the constitutive TEF promoter (pTEF). The mature sequence of wild type Lip2 from species of the *Yarrowia* clade and CAHI were integrated downstream either the PrePro-Lip2 secretion signal of *Y*. *lipolytica* or their own PrePro secretion signal (Table 2).

**Table 2:** Strains used for lipase expression in *Y*. *lipolytica*.

CLIB number	Lipase expressed	Genotype	Reference
CLIB 1677 = JMY1212		*MATA ura3-302 leu2-270-LEU2-zeta xpr2-322 Δlip2 Δlip7 Δlip8 Leu* ^*+*^, *Ura* ^*−*^	[40]
CLIB 1678	*YlLIP2*	JMY1212 *+ pTEF-YlLIP2-URA3ex*	[19]
CLIB 1679	*YlLIP2-*V232S	JMY1212 *+ pTEF-YlLIP2-URA3ex*	[18]
CLIB 1680	*YlLIP2-*D97A-V232F	JMY1212 *+ pTEF-YlLIP2-URA3ex*	[52]
CLIB 1681	*YpLIP2a*	JMY1212 *+ pTEF-YpLIP2a-URA3ex*	This study
CLIB 1682	*YpLIP2a*	JMY1212 *+ pTEF-YlPrePro-YpLIP2a-URA3ex* *	This study
CLIB 1683	*YpLIP2a-*V232S	JMY1212 *+ pTEF-YpLIP2a-URA3ex*	This study
CLIB 1684	*YpLIP2a-*D97A-V232S	JMY1212 *+ pTEF-YpLIP2-URA3ex*	This study
CLIB 1685	*YpLIP2b*	JMY1212 *+ pTEF-YpLIP2b-URA3ex*	This study
CLIB 1686	*YpLIP2b*	JMY1212 *+ pTEF-YlPrePro-YpLIP2b-URA3ex* *	This study
CLIB 1687	*YpLIP2c*	JMY1212 *+ pTEF-YpLIP2c-URA3ex*	This study
CLIB 1688	*YpLIP2c*	JMY1212 *+ pTEF-YlPrePro-YpLIP2c-URA3ex* *	This study
CLIB 1689	*YpLIP2d*	JMY1212 *+ pTEF-YpLIP2d-URA3ex*	This study
CLIB 1690	*YpLIP2d*	JMY1212 *+ pTEF-YlPrePro-YpLIP2d-URA3ex* *	This study
CLIB 1691	*YpLIP2e*	JMY1212 *+ pTEF-YpLIP2e-URA3ex*	This study
CLIB 1692	*YpLIP2e*	JMY1212 *+ pTEF-YlPrePro-YpLIP2e-URA3ex* *	This study
CLIB 1693	*YgLIP2*	JMY1212 *+ pTEF-YgLIP2-URA3ex*	This study
CLIB 1694	*YgLIP2*	JMY1212 *+ pTEF-YlPrePro-YgLIP2-URA3ex* *	This study
CLIB 1695	*YyLIP2*	JMY1212 *+ pTEF-YyLIP2-URA3ex*	This study
CLIB 1696	*YyLIP2*	JMY1212 *+ pTEF-YlPrePro-YyLIP2-URA3ex* *	This study
CLIB 1697	*YaLIP2*	JMY1212 *+ pTEF-YaLIP2-URA3ex*	This study
CLIB 1698	*YaLIP2*	JMY1212 *+ pTEF-YlPrePro-YaLIP2-URA3ex* *	This study
CLIB 1699	*YhLIP2a*	JMY1212 *+ pTEF-YhLIP2a-URA3ex*	This study
CLIB 1700	*YhLIP2a*	JMY1212 *+ pTEF-YlPrePro-YhLIP2a-URA3ex* *	This study
CLIB 1701	*YhLIP2b*	JMY1212 *+ pTEF-YyLIP2b-URA3ex*	This study
CLIB 1702	*YhLIP2b*	JMY1212 *+ pTEF-YlPrePro-YhLIP2b-URA3ex* *	This study

CLIB numbers refer to the International Center for Microbial Resources, CIRM-Levures (http://www7.inra.fr/cirmlevures/)

*YlPrePro-Yx*LIP2* corresponds to the fusion of the *Y*. *lipolytica LIP2* PrePro region with the region of the expressed lipase corresponding to the mature coding region.

For constructions using the PrePro-Lip2 secretion signal of *Y*. *lipolytica*, forward and reverse primers were designed to amplify the mature sequence of each Lip2 gene from species of the *Yarrowia* clade and CAHI, and to introduce *Bsrg*I and *Avr*II restriction sites. The PCR product obtained from genomic DNA was digested with these restriction enzymes and ligated to the plasmid JMP62-*URA3*ex-pTEF digested with the same restriction enzymes and gel purified. For constructions using their own PrePro signal secretion, the In-Fusion HD Cloning Plus kit from Clontech (Mountain View, CA, US) was used. Primers were designed to amplify the complete sequence of each Lip2 gene, including the PrePro secretion signal, and at each extremity, to introduce 15 bases homologous to those of the vector linearized by PCR amplification using both primers aJMP62TEFPPf and aJMP62TEFLip2YLr.

Variants V232S and D97A-V232F of YpLip2a were constructed using site directed mutagenesis. PCR amplification was performed using plasmid JMP62-YpLip2a as template and overlapping primers containing the desired mutation. PCR reaction was subsequently digested by *Dpn*I and directly transformed in *E*. *coli* DH5α strain.

All primers were purchased from Eurogentec (Angers, France) and the sequences are listed in S3 Table. For all constructions, sequences were checked by DNA sequencing (GATC-Biotech, Konstanz, Germany) to ensure that no mutation was introduced during the PCR. *E*. *coli* DH5α (Life Technologies, Carlsbad, CA, US) was used for vector construction and amplification. The plasmids were digested with *Not*I and used for transformation of strain JMY1212 (MATA ura3–302 leu2–270-LEU2-zeta xpr2–322 Δlip2 Δlip7 Δlip8) [40] by the lithium acetate method as described previously [41]. The strains constructed in this study were deposited at the International Center for Microbial Resources, CIRM-Levures (http://www7.inra.fr/cirmlevures/).

### Production of recombinant enzymes in Y. lipolytica

Erlenmeyer flasks (500 mL) containing medium Y_1_T_2_O_3_ (50 mL total) made of yeast extract (10 g/L), bactotryptone (20 g/L), and oleic acid (30 g/L), buffered with phosphate buffer (100 mM, pH 6.8) were inoculated with cells pre-grown in YPD containing yeast extract (10 g/L), bactopeptone (10 g/L), and glucose (10 g/L) at an initial cell density of OD_600_ = 0.2. Stock solution containing oleic acid (200 g/L) and Tween40 (5 g/L) was subjected to sonication three times for 1 min each on ice for emulsification purposes. Cells were incubated at 28°C for 24 h until complete consumption of oleic acid. The cells were centrifuged at 10,000 rpm for 10 min, and supernatants were directly used in reactions.

### SDS-PAGE analysis

Samples were loaded on 10% NuPAGE Tris-acetate sodium dodecyl sulphate-polyacrylamide electrophoresis gel (Invitrogen, Cergy Pontoise, France). After migration, the gel was subjected to silver staining.

### Hydrolysis of p-nitrophenyl butyrate

Lipase activity in the culture supernatant was determined by monitoring the hydrolysis of *p*-nitrophenyl butyrate (*p*NPB) into butyric acid and *p*-nitrophenol (*p*-NP) [42]. The method was optimised using 2-methyl-butan-2-ol (2M2B) as solvent to solubilize *p*-NPB [40]. Lipase activity was measured in 96-well microplates with 20 μL of the supernatant containing properly diluted lipases, 175 μL of a 100 mM phosphate buffer pH 7.2 containing 100 mM NaCl and 5 μL of 40 mM pNPB in 2M2B. Activity was measured by monitoring absorbance at 405 nm at 25°C for 10 min using the VersaMax tunable microplate reader apparatus (Molecular Devices, Rennes, France). One unit of lipase activity was defined as the amount of enzyme releasing 1 μmol of pNP per min at 25°C and pH 7.2.

### Resolution of (R, S) 2-bromo phenylacetic acid octyl esters

Hydrolysis was carried out in 2 mL Eppendorf tubes containing a biphasic medium composed of 0.75 mL dried decane containing the ester (50 mM) and 0.75 mL of the aqueous enzymatic solution. The mixture was shaken in a Vortex Genie 2 (Dutscher, Brumath, France). Reactions were performed at 25°C. After phase separation by centrifugation (50 μL diluted in 500 μL hexane), the progress of the reaction was monitored by taking samples at regular intervals.

### HPLC analysis

The HPLC device was equipped with a chiral column: Chiralpack OJ (25 cm x 4.6 mm) (Daicel Chemical Industries Ltd, Japan) connected to a UV detector (at 254 nm). A flow rate of 1.0 mL/min and a 40°C column temperature were used. The mobile phase was composed of a mixture of *n-*hexane/isopropanol (80:20 v/v).

### Determination of the enantiomeric excess, conversion and enantioselectivity

The enantioselectivity (*E-*value) was the ratio of the initial rate:

E = (viS / viR) for a S enantioselectivity

E = (viR / viS) for a R enantioselectivity.

ViS and viR are the initial rates of the S and R enantiomer consumption, respectively. The initial rate was determined by linear regression with a maximum conversion of substrate of less than 15%.

## Results

### A large family of lipases in the *Yarrowia* clade

*Y*. *lipolytica* is known to have undergone a lipase family expansion in a different way from most *Saccharomycotina* species. Indeed, 16 genes were recovered from the complete genome sequence [43]. We searched for homologues in the other sequenced species and found similar expansion in *Y*. *galli* (YAGA) with 14 genes. Expansion was more limited in the other species, with 9 genes in *Y*. *yakushimensis* (YAYA; 7 + 2 pseudogenes) and *Y*. *phangngensis* (YAPH; 8 + 1 pseudogene), 10 genes (9 + 1 pseudogene) in *Y*. *alimentaria* (YAAL), and only three genes in the out-group species *Candida hispaniensis* (CAHI). Thus, the set of LIP genes, the so called LIP family, is composed of 61 members of which 4 are pseudogenes (sequences are listed in S1 Table). The two YAYA pseudogenes were shown to be full-length copies inactivated by a frameshift (YAYA0S2-34200g) or a stop in frame (YAYA0S4-04016g). The YAPH and YAAL pseudogenes corresponded to truncated copies (YAPH0S3-02454g and YAAL0S2-06656g). Full-length proteins ranged from 299 to 429 amino acids, the longest being YALI0D09064g (= YlLip11) with a putative N-terminal extension of 85 amino acids. Overall, the N-ter of the proteins was poorly conserved whereas the rest of the alignment was rather well conserved covering about two thirds of the proteins. All the proteins in this region harbored the conserved serine, aspartic acid, and histidine catalytic triad characteristic of the α/β hydrolase with the catalytic nucleophile serine located in the highly conserved pentapeptide GHS[LFM]G [1]. This finding confirms that all of these genes encode lipases. The most closely related lipases already published in other species are three lipases of *Candida deformans*, which also belongs to the *Yarrowia* clade [6]. The set of LIP genes, including those reported in *C*. *deformans*, but with the exception of *C*. *hispaniensis* LIP9, forms a monophyletic group sharing 32 to 97% identity. Outside this group, the proteins which share the highest level of identity correspond to putative lipases of *Wickerhamomyces ciferii* and *Kuraishia capsulata* with 31–33% identity and 51–52% similarity, respectively. Filamentous ascomycetes such as *Thermomyces lanuginosa*, *Penicillium*, *Blumeria*, or *Aspergillus* species as well as early-diverging fungus such as *Mucor miehei* or *Rhizopus niveus* contain LIP2 homologues which share about 25–30% identity and 40–50% similarity. A putative lipase was also identified in *Sacharomycetaceae*. This putative lipase in *Saccharomyces cerevisiae*, called Lih1 and encoded by YJR107W, shares 26% identity and 44% similarity with YlLip2, its closest relative in *Y*. *lipolytica*. Lih1 and its counterparts in other Hemiascomycetes may derive from a common LIP ancestor.

### Reconstruction of an evolutionary scenario of the lipase family

**Fig 1:**
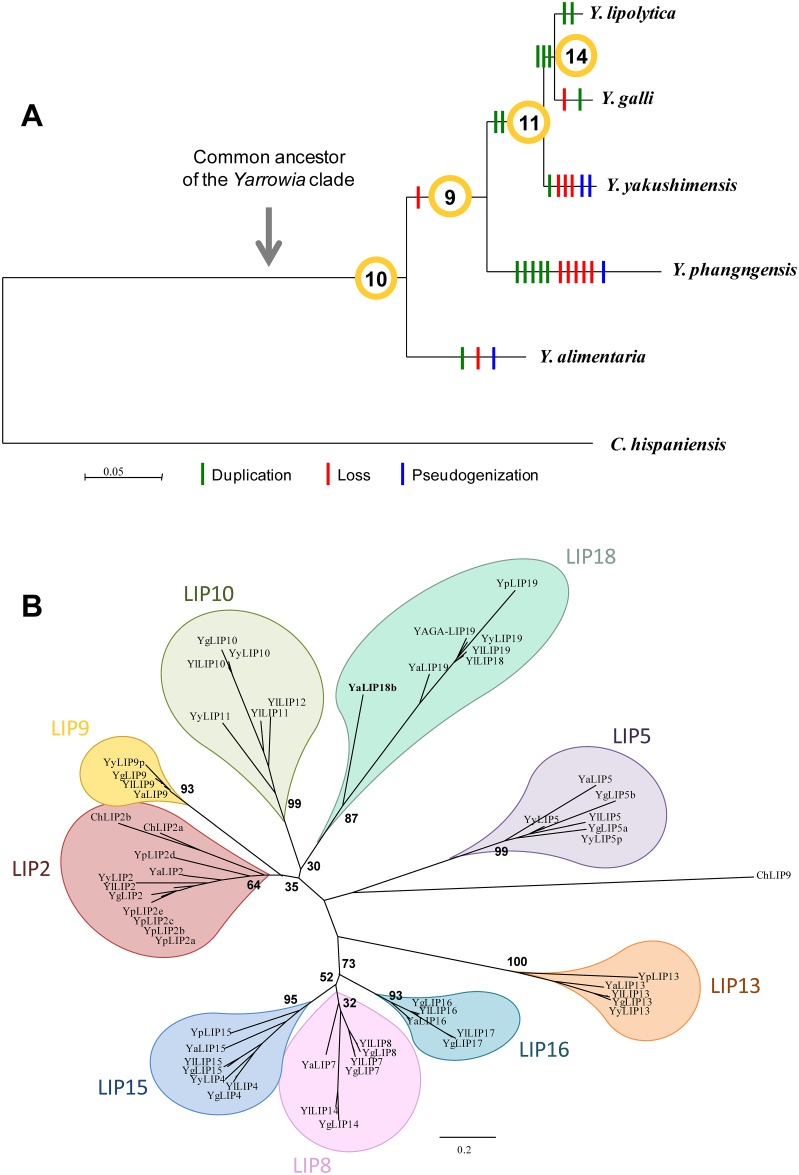
Phylogeny and evolutionary scenario of the lipase genes in the *Yarrowia* clade. (A) Evolutionary events are placed on the species tree constructed from the concatenation of 912 proteins (398959 residues). Duplications, losses and pseudogenizations are depicted as vertical green, red and blue lines, respectively. The number of ancestral genes at each ancestral node is given within yellow circles. (B) Phylogeny of the lipases from the *Yarrowia* clade and *C*. *hispaniensis*. The tree is based on the alignment of 59 lipases, further cleaned with Gblocks (133 amino acid residues). The two full-length pseudogenes YyLIP9p and YyLIP5p are included in the tree, whereas the truncated pseudogenes are not. Groups of lipases derived from the 10 ancestral copies are depicted as teardrops of different colours. The tenth ancestral lipase, called LIP18b, is present only in YAAL (YaLIP18b in bold, included in the LIP18 group). Bootstrap values are represented at the most ancestral nodes.

To reconstruct the evolution of the lipase family, CAHI was used as an out-group and the common ancestor of the clade was placed after the divergence of CAHI, just before the branching of YAAL (Fig 1A). To determine the phylogenetic relationships between the different lipases of the 6 species, an amino acid alignment was performed for the 59 full-length genes. The phylogeny based on this alignment made it possible to separate the proteins into 9 monophyletic groups with at least 4 members (Fig 1B). In each group, we compared the phylogeny of the genes and the species, and we used synteny conservation to reconstruct the evolutionary scenario and thus to determine the number and the relative location of the ancestral lipases. This strategy showed that 15 duplications, 11 losses and 4 pseudogenizations may have occurred during the evolution of 10 ancestral genes (LIP2, LIP4, LIP5, LIP8, LIP9, LIP10, LIP13, LIP16, LIP18 and LIP18b). With on average more than 3 events per ancestral gene, the lipase family is thus highly dynamic in this clade with more duplications than losses. Assuming that CAHI has only three lipases, the family expansion may predate the divergence of the clade and is still underway in the most closely related species of YALI. Two duplications occurred before the divergence of YAYA/YAGA/YALI and three before the divergence of YAGA/YALI. All the other duplication events are species-specific (Fig 1A). Finally in YALI, which has the highest number of lipases in the clade, LIP15 derived from the duplication of LIP4, LIP7 and LIP14 from LIP8, LIP11 and LIP12 from LIP10, LIP17 from LIP16 and LIP19 from LIP18, but only two duplications are species-specific (LIP12 and LIP19). The fact that 10 out of 11 losses are species-specific also contributed to the lipase family expansion. The 11th loss occurred before the divergence of YAPH and allowed the definition of a tenth ancestral lipase (LIP18b), absent from YALI and restricted to YAAL (Fig 1B). We did not find any evidence for a putative PrePro domain in YaLIP18b, suggesting that the protein is not secreted. However, it would be interesting to investigate its intracellular activity and substrate specificity, if any.

**Fig 2:**
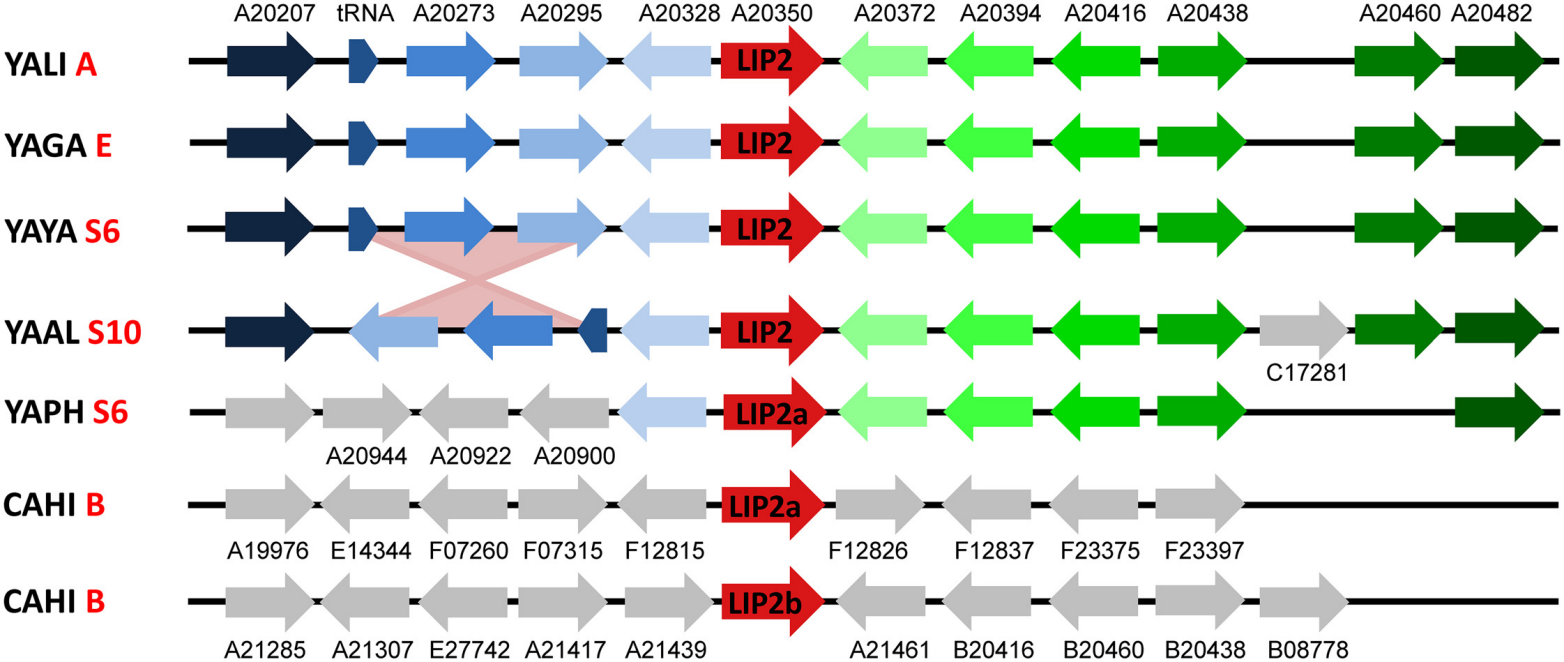
Synteny conservation between LIP2 genes. Orthologous genes are represented by arrows of the same colour. The name of the *Y*. *lipolytica* ortholog is abbreviated above the first line of genes or under the non-orthologous genes that are depicted in grey. The inversion event that occurred between YAYA S6 and YAAL S10 is represented with two pink inverted triangles. Chromosome or scaffold abbreviations on the left correspond to *Y*. *lipolytica* chromosome A (YALI A), *C*. *galli* chromosome E (YAGA E), *Y*. *yakushimensis* scaffold 6 (YAYA S6), *C*. *alimentaria* scaffold 10 (YAAL S10), *C*. *phangngensis* scaffold 6 (YAPH S6) and *C*. *hispaniensis* chromosome B (CAHI B).

The number of evolutionary events was not uniformly distributed among the phylogenetic groups of lipases. For instance, no event occurred in the LIP13 group and all the LIP13 genes are still conserved in synteny. On the contrary, the LIP2 gene has undergone multiple evolutionary events. Six independent duplications occurred, five in YAPH and one in YAAL. In both species, one non-ancestral copy is pseudogenized. However, in the 5 species which make up the *Yarrowia* clade, the ancestral copy of LIP2 is still conserved in synteny (Fig 2).

### Expression patterns are conserved only in the LIP2 family

**Fig 3:**
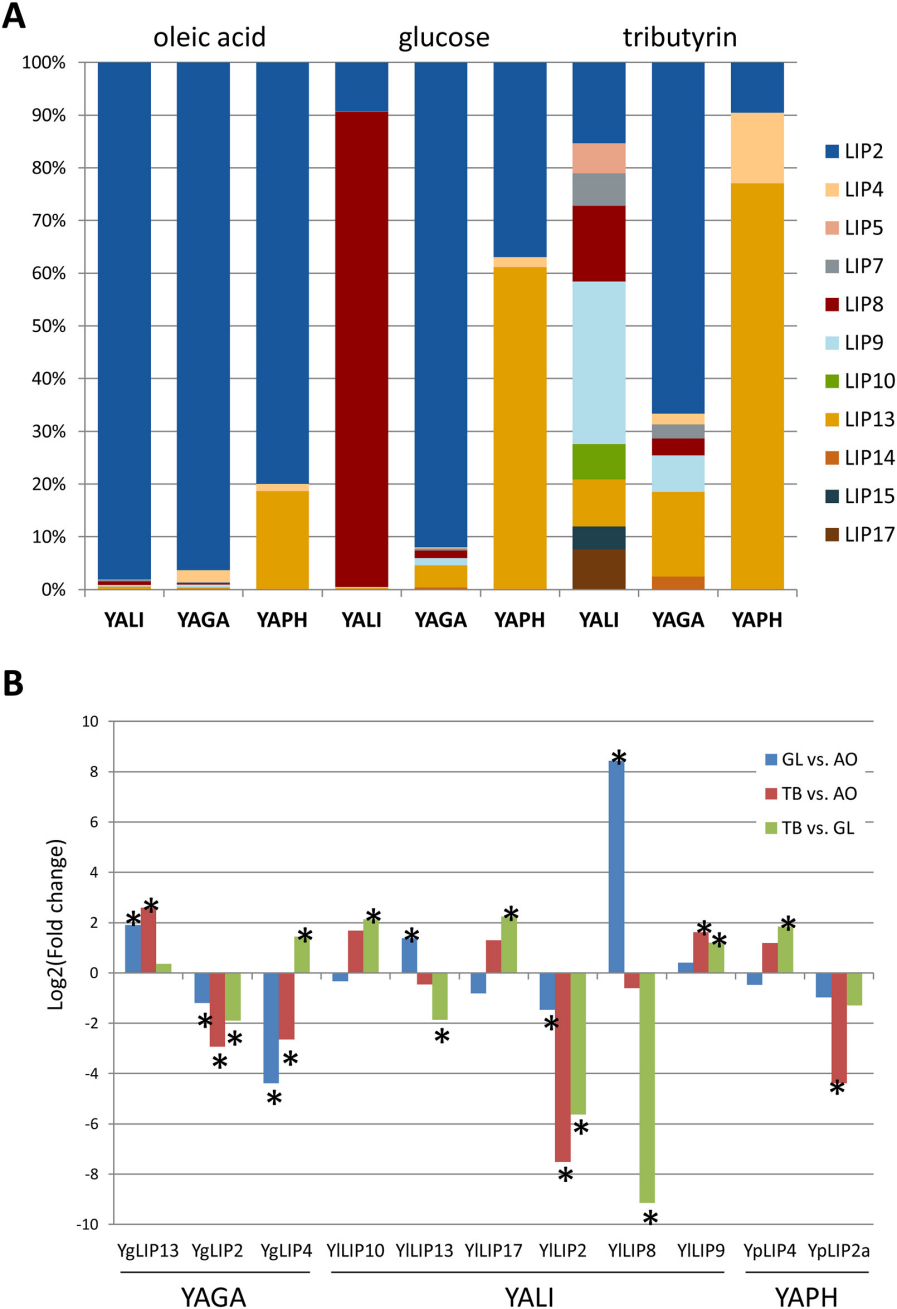
Relative expression of lipases according to growth media in YALI, YAGA and YAPH. (A) The number of reads has been normalized by the length of the CDS for each gene and expressed as RPK (reads per kb). LIP name correspond to orthologous genes. In the case of *C*. *phangngensis*, the LIP2 corresponds to LIP2a which is the syntenic ortholog. (B) The fold change (FC) between each couple of conditions is provided for each expressed lipase of YALI, YAGA and YAPH with a significant adjusted P-value in at least one couple of conditions. Stars indicate fold change with a significant adjusted P-value (cut-off of adjP-value is 1.e-3 for YALI and 5.e-3 for YAGA and YAPH). GL: glucose, AO: oleic acid, TB: tributyrin.

Transcriptomic analysis through a RNA-Seq strategy was conducted on YALI, YAGA and YAPH. These species have at least 9 different genes, however only half of them (19 out of 39) were transcribed in the culture conditions of our study, i.e. minimal medium plus oleic acid, tributyrin or glucose. No reads were obtained in the RNA-Seq data for 7 lipases of YALI and 7 of YAGA. In YAPH, none of the LIP2-like lipases except LIP2a which is the syntenic ortholog of YlLIP2 and thus the ancestral copy, were transcribed, neither the YpLIP18 lipase. Thus, only 9 lipases in YALI, 7 in YAGA and 3 in YAPH were considered to be transcriptionally active (S2 Table). Their relative expression differed with the growth media. In the three strains, YlLIP2, YgLIP2 and YpLIP2 were the lipases most frequently expressed in oleic acid with more than 95% of the lipase transcripts in YALI and YAGA, and about 80% in YAPH (Fig 3A). Surprisingly, in YALI the major lipase in glucose was YlLIP8 whereas it was still YgLIP2 in YAGA. In YAPH, the expression of YpLIP13 prevailed in both glucose and tributyrin. In these media, there was no predominant lipase in YALI. Among the 19 expressed lipases, at least 11 lipases appeared to be regulated, i.e. they showed statistically significant difference in levels of expression in at least one media comparison (see the adjusted P-value in S2 Table and Fig 3B). The three expressed LIP2 lipases showed the same pattern of expression regulation. These lipases were expressed in the three media and were mainly induced on oleic acid and slightly less on glucose than on tributyrin. Conservation of the pattern of regulation between orthologous lipases is not a general rule. Indeed, YlLIP4 was not expressed in any condition; YpLIP4 was mainly induced on tributyrin and YgLIP4 on oleic acid. Similarly, YpLIP13 was expressed in all three conditions; YlLIP13 was induced on glucose and YgLIP13 on oleic acid. In YALI, four additional lipases were shown to be regulated by growth conditions (LIP8, LIP9, LIP10 and LIP17); none of these lipases was found in YAPH but were present in YAGA. LIP10 and LIP17 were induced by tributyrin in YALI and not expressed in YAGA. LIP9 was induced by tributyrin in YALI and was always expressed in YAGA.

### A promising strong promoter inducible by glucose

The most remarkable regulation was that of YlLIP8 which was highly induced by glucose with fold change values of 360 and 562 for glucose versus oleic acid and tributyrin, respectively. A comparison of the level of expression between YlLIP8 and all other protein-coding genes of *Y*. *lipolytica* showed that YlLIP8 is one of the 25 most frequently expressed genes on glucose (read counts per kb in both glucose replicates). This gene is almost never expressed on oleic acid or tributyrin. Its expression pattern in our conditions resembled that of the XPR2 promoter (pXPR2) from YALI0F31889g [44]. pXPR2 is a strong inducible promoter with a complex induction [45], which has encouraged people to use hybrid chimera promoters based on the XPR2 upstream activation sequences [46, 47]. Assuming that pLIP8 has the same expression and specificity as pXPR2, it may be an interesting alternative promoter with less complex requirements for induction, and deserves further investigation.

### Structural characteristics of the LIP2 subfamily

The LIP2 gene is the only lipase gene present in all species of the clade and in CAHI. This gene has undergone the highest number of duplications and the ancestral copy is the most frequently expressed lipase in oleic acid in YALI, YAGA and YAPH. All these findings mean this subfamily, the so- called LIP2 family, would be an interesting group of genes in which to investigate their respective activity and enantioselectivity in relation with their sequence features.

**Fig 4:**
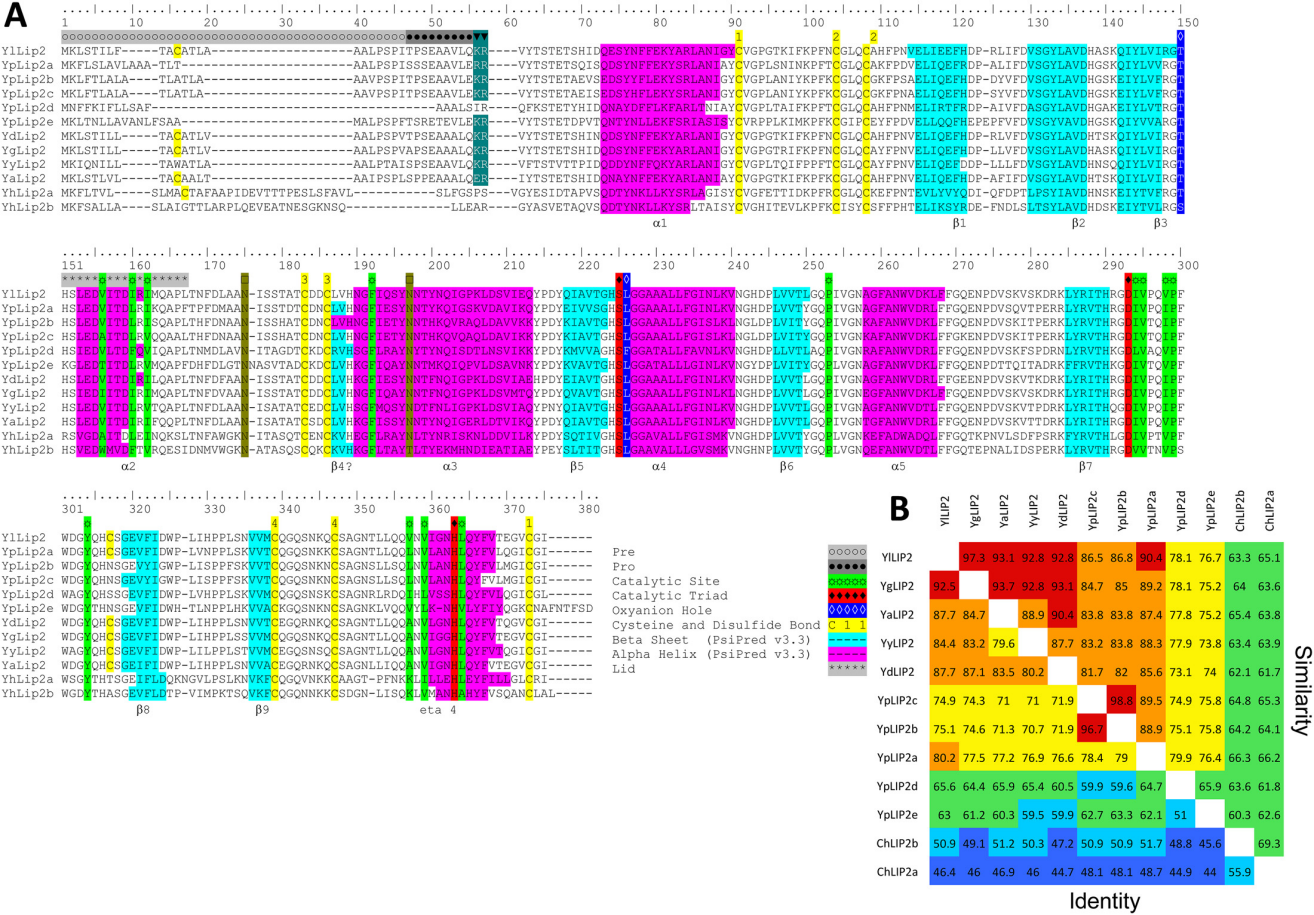
Amino acids sequence comparison of lipases from the LIP2 family. (A) Amino acids sequence alignment. Residues forming part of α helices and β strands are coloured in magenta and cyan, respectively. The three catalytic residues are coloured in red, the two catalytic residues of the oxyanion hole in dark blue, the cysteines are yellow-coloured and the lid is in grey above the alignment. The α/β hydrolase fold of YlLip2 is from Fig 3 of Bordes et al. (Bordes et al. 2010). β-sheet are numbered according to the current nomenclature (Ollis et al. 1992). The YdLip2 corresponds to YdLIP1 (AJ428393.1; Bigey et al. 2003). (B) Pairwise sequence comparison. Similarity and identity are calculated along the whole pariwise alignment and provided as percentage. Colours are according to the level of conservation: below 50% (blue), 50–60% (cyan), 60–70% (green), 70–80% (yellow), 80–90% (orange) and 90–100% (red).

**Fig 5:**
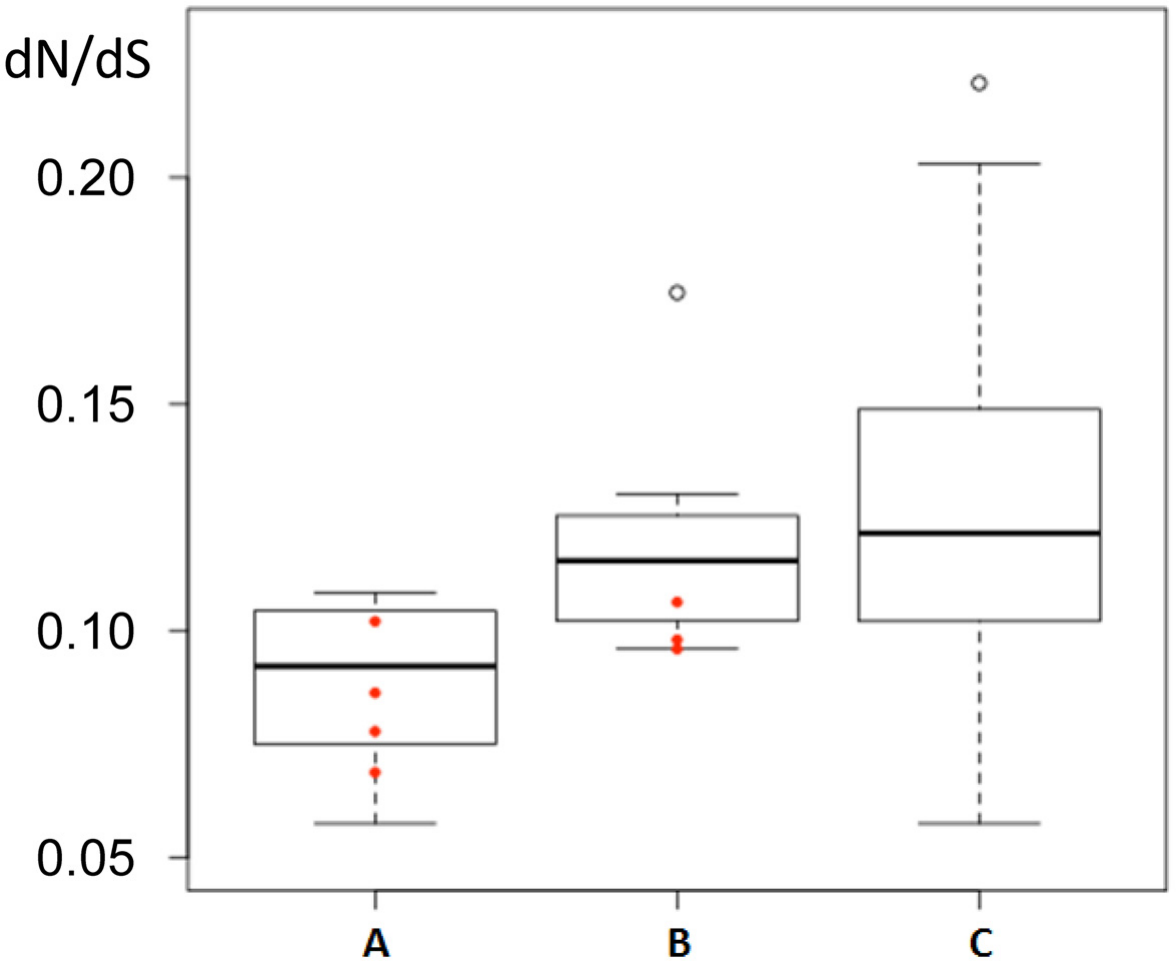
Box-plot of the dN/dS ratio estimated for the LIP2 genes based on pairwise alignments. Different sets of genes were compared: pairs of ancestral LIP2 genes (A), pairs of YpLIP2 genes (B) and ancestral versus non-ancestral LIP2 genes (C). Red dots represent the dN/dS values involvingYpLIP2a.

The sequence alignment of the 10 newly identified Lip2 lipases with YlLIP2 and the Lip2 homologue previously identified in *Y*. *deformans* [6] is shown in Fig 4A. Compared to YlLip2, the YgLip2, YdLip2 and YyLip2a lipases present the highest level of conservation with 87.7 to 92.5% pairwise identity and 92.8 to 97.3% similarity, which is congruent with the species phylogeny. In contrast, the higher conservation of YlLip2 with YaLip2 (87.7% identity and 93.1% similarity) rather than with YpLip2a, which is the ancestral copy in YAPH, is surprising (Fig 4B). As both genes are still conserved in synteny (Fig 2), the high level of sequence conservation of YaLip2 may be due to a horizontal gene transfer followed by a conversion event. A possible alternative explanation is that YpLip2a diverged more rapidly due to the presence of 5 duplicated copies in YAPH, even though, among the YAPH LIP2 lipases, YpLip2a, which is the ancestral copy, remains the most conserved. To test this alternative hypothesis, we quantified the selective constraints on the LIP2 genes by estimating the ratio of per site non-synonymous (dN) to synonymous (dS) substitution rates (dN/dS). As shown in Fig 5, all LIP2 genes were shown to be under purifying selection, i.e. the dN/dS ratio was less than 1. The pressure acting on non-ancestral LIP2 genes was much lower than on ancestral LIP2 pairs, which might be due to a relaxation of the purifying selection on duplicated copies. Although multiple copies exist in YAPH, the ancestral YpLIP2a gene was seen to be under the same purifying selection pressure as ancestral copies in the other genomes where no LIP2 duplications occurred. This finding supports the first hypothesis of a horizontal transfer at the locus in YAAL.

Also noteworthy is the fact that YpLIP2b and YpLip2c are almost identical with only 11 amino acid differences, suggesting that one is a duplicated copy of the other. In contrast, the two LIP2 copies in CAHI shared only 55.9% identity and 69.3% similarity, probably resulting from a long evolution. Sequencing the LIP2 locus of additional species of the *Yarrowia* clade, especially those which have been described very recently, will provide clues to decipher the strange phylogeny of the LIP2 gene [48–50].

The secondary structure pattern appeared to be highly conserved among all 12 enzymes, with a perfect alignment of α-helices and β-strands. The classical signature GHSLG of lipases from the filamentous fungi superfamily, which includes the catalytic serine, was seen to be almost conserved. The only exception was YpLip2d from *Y*. *phangngensis* in which the leucine next to the catalytic serine was replaced by a phenylalanine. The two other amino acids of the catalytic triad, namely Asp230 and His289 in YlLip2, were perfectly aligned located after strand β7 and β9, respectively. The two residues forming the oxyanion hole, which stabilize the tetrahedral intermediate formed during the reaction mechanism, were aligned and identical. The first residue belongs to the “GX” type lipase according to the classification proposed by Pleiss *et al*., which bears specificities for medium and long chain fatty acids, X being a threonine in all these lipases [51]. The second residue, located next to the catalytic serine, was a leucine, except for the previously mentioned YpLip2d with a phenylalanine.

Disulphide bonds play an essential structural role in proteins by stabilizing their tertiary structure. YlLip2 is stabilised by four disulphide bonds (Cys30-Cys299, Cys43-Cys47, Cys120-Cys123, Cys265-Cys273), and contains one free cysteine (Cys244) [35]. All the lipases from the LIP2 family appeared to be stabilised by the same four disulphide bonds. The extra cysteine, Cys244 reported in YlLip2, was present in all ancestral LIP2 lipases of the clade, i.e. in YpLip2a, YgLip2, YyLip2 and YaLip2, but not in YhLip2a,b. This extra cysteine has been shown to be detrimental to enzyme thermostability in YlLip2, leading to intramolecular disulphide bonds during denaturation and protein aggregation [19].

One specificity of lipases is the fact that catalytic activity is strongly dependent on the structural rearrangement of a mobile sub-domain, called the lid. The lid was seen to block the active site in the closed form of the enzyme and to move away in presence of a hydrophobic interface, and had an open conformation when the active site became accessible to the substrate. Generally, the sequence of the lid (from L91 to I100 in YlLip2) of the different lipases was similar. YhLip2a and YhLip2b had the most dissimilar lid sequence.

The substrate binding site of YlLip2 appeared as a hydrophobic crevice located at the protein surface, with a catalytic triad exposed to the solvent, like in all mucorales lipases. The hydrophobic crevice consisted of T88, V94, I98, I100, F129, L163, P190, V232, V235, P236, Y241 in YlLip2. The scissile fatty acid of a triglyceride is assumed to bind to this hydrophobic crevice. The sn-2 substituent is thought to bind to the hydrophobic dent formed by I231, V283, V285 and L290. Table 3 lists the amino acids located at these positions for the 12 Lip2 lipases. Amino acids 129, 190, 232, 236 and 241 were conserved in the 15 positions. The first amino acid of the oxyanion hole in position 88 was a threonine, except in YhLip2b, where it was a serine like in other mucorales lipases such as *Mucor miehei* and *Thermomyces lanuginosa* [19]. The second amino acid of the oxyanion hole was a leucine except for YpLip2d where it was a phenylalanine. Amino acids in positions 98 and 100, located in the lid, and in 231, 235, 285 and 290, were all hydrophobic amino acids (V, L, I, A or F). Positions 94 and 283 presented the highest variability. In position 94, hydrophobic amino acids are generally encountered, with a tryptophan in YhLip2b, but a threonine was found in YpLip2e. The amino acid in position 283 was a hydrophobic residue except for YhLip2a and YhLip2b, where it was a lysine, a positively charged amino acid.

**Table 3:** Amino acids involved in the hydrophobic crevice and hydrophobic dent.

	YlLip2	YpLip2a	YpLip2b	YpLIP2c	YpLip2d	YpLip2e	YgLip2	YdLip2	YyLip2	YaLip2	YhLip2a	YhLip2b
**88**	T	T	T	T	T	T	T	T	T	T	T	**S**
**94**	V	V	V	A	V	**T**	I	V	V	V	A	**W**
**98**	I	L	L	L	F	L	I	I	L	I	F	L
**100**	I	I	I	V	V	V	I	I	V	I	I	V
**129**	F	F	F	F	F	F	F	F	F	F	F	F
**163**	L	L	L	L	**F**	L	L	L	L	L	L	L
**190**	P	P	P	P	P	P	P	P	P	P	P	P
**231**	I	I	I	L	I	I	I	I	I	I	I	I
**232**	V	V	V	V	V	V	V	V	V	V	V	V
**235**	V	I	I	I	V	V	I	I	V	V	V	V
**236**	P	P	P	P	P	P	P	P	P	P	P	P
**241**	Y	Y	Y	Y	Y	Y	Y	Y	Y	Y	Y	Y
**283**	V	L	L	L	I	V	A	A	A	A	**K**	**K**
**285**	V	V	V	V	L	L	V	V	V	V	I	V
**290**	L	L	L	L	L	V	L	L	L	L	L	A

Finally, YlLip2, as an excreted protein, was expressed in the form of a PrePro enzyme precursor (Fig 4A and S1 Fig). The pre region is composed of 22 amino acids with four X-Ala or X-Pro dipeptides, substrates of a diamino peptidase which cleaves after Ile22 [12]. It is followed by a short pro region of 12 aa finished by a Lys-Arg dipeptide, substrate of the *KEX2*-like endopeptidase encoded by the *XPR6* gene in *Y*. *lipolytica* [12]. YpLip2d had no clear PrePro region. YhLip2a and b PrePro regions diverged from the YlLip2 PrePro region. As shown on the alignment of the 9 most closely related PrePro regions to that of YlLip2, two X-Arg dipeptides were present at the end of most of these PrePro regions, except in YaLip2, YdLip2, YgLip2 and YpLip2a, where only the Arg-Arg dipeptide was observed (S1 Fig). Two X-Pro were also present in the PrePro of these 9 lipases except in YyLip2 where the second dipeptide, Ser-Pro, was replaced by a Thr-Ala.

### Expression of LIP2 genes in *Y*. *lipolytica*

All LIP2 genes except YdLIP2 were cloned in *Y*. *lipolytica* strain JMY1212 [40] under the control of the constitutive TEF promoter from *Y*. *lipolytica*. Two strategies were used: either the entire genes were cloned in the presence of their own PrePro sequences or the part of the genes coding for the mature proteins were cloned behind the PrePro region of YlLip2. Hydrolytic activities of *p*Nitro Phenyl Butyrate (*p*NPB) were measured and are presented in Table 4.

**Table 4:** *p*-nitrophenyl butyrate hydrolysis activity of secreted lipases using either the PrePro region of YlLip2 or the original PrePro region of each lipase.

Enzyme	PrePro YlLip2*	PrePro YxLip2*
YlLip2	57.5	57.5
YpLip2a	19.4	23.7
YpLip2b	13.2	24.0
YpLip2c	23.3	25.7
YpLip2d	0	0
YpLip2e	0.2	0.9
YgLip2	16.8	12.9
YyLip2	4.3	5.8
YaLip2	58.7	50.6
YhLip2a	0.5	0.7
YhLip2b	0	0

Experiments were performed in triplicate.

*μmol of *p*-nitrophenol liberated per minute.

Seven lipases, YlLip2, YaLip2, YpLip2a, YpLip2b, YpLip2c, YgLip2 and YyLip2, presented significant activities whatever the strategy used, suggesting that the origin of the PrePro region has no clear influence on the result. The other lipases, YpLip2d, YpLip2e, YhLip2a and YhLip2b, showed no or very low activity when expressed in their own PrePro region. Except YpLip2e, all had a different N-terminal region from the PrePro of other LIP2 members, but even when the latter were replaced by the PrePro region of YlLip2, no or very little activity was detected. This suggests that either the protein was not correctly secreted in the medium, or degraded rapidly, or that the secreted protein no longer showed lipase activity. The presence of a secreted protein was checked on a protein gel. This experiment showed that all 7 active enzymes presented similar expression, whereas in the case of no or low activity, no protein expression was detected whatever the PrePro system used (S2 Fig). This absence of protein is surprising and may reflect a problem in the coding sequence, as in each case two different proteins were produced, one with YlLip2 PrePro and one with the native PrePro. For YpLip2d, we additionally constructed the F165L variant with YlLip2 PrePro. Indeed, YpLip2d presented extra specificity, as it had a bulky atom in the oxyanion hole, a phenylalanine, instead of a leucine. This F165L variant was constructed but the activity was not restored, suggesting that the absence of activity is not solely due to this amino acid substitution.

Interestingly, in YAPH, three Lip2 lipases showed significant enzymatic activity under the TEF promoter in YALI, but we showed that only YpLip2a was expressed in YAPH under its own promoter in the conditions used for the RNA-Seq analysis (see section Expression patterns are conserved only in the LIP2 family). Indeed, the genes coding for YpLip2b and YpLip2c were not transcribed in the wild type strain but were well expressed when controlled by the TEF promoter in YALI.

### Enantioselectivity of the LIP2 subfamily

The enantioselectivity of 11 lipases from the LIP2 family was tested during the resolution by hydrolysis of 2-bromo-arylacetic acid esters (Table 5). YpLip2a was clearly the most efficient lipase from both kinetic and selectivity points of view. It displayed 2–fold enhanced activity toward the *S*-enantiomer whereas its activity toward the *R*-enantiomer was about 5 times lower than that obtained with the YlLip2. As a consequence, enantioselectivity increased remarkably, by almost one order of magnitude, from an *E-*value of 3 to 30, compared to other lipases.

**Table 5:** 2-bromo-phenylacetic acid ethyl ester hydrolysis activity of wild-type lipases of the LIP2 family and of variants of YpLip2a.

Enzyme	YlLip2	YpLip2a	YpLip2b	YpLip2c	YgLip2	YyLip2	YaLip2	YpLip2a V232S	YpLip2a D97A V232F
(S)-Initial rate[a]	2.4	4.6	1.02	1.29	2.41	3.2	2.16	11.6	0
(R)-Initial rate[a]	0.72	0.153	0.53	0.52	0.52	0.58	0.60	0	4.8
*E*-value ^[b]^	3.3 (*S*)	30.1 (S)	1.9 (S)	2.5 (S)	4.6 (S)	5.5 (S)	3.6 (S)	> 200 (S)	> 200 (R)

^[a]^ μmol of 2-bromo-phenylacetic acid liberated per hour and mL of enzyme.

^[b]^ E-value = viS/viR or viR/viS according to enantiomer preference; viR, viS: initial rates.

Position 97 and mainly position 232 were identified as crucial for distinguishing between enantiomers. However, these two positions are perfectly conserved with a valine and an aspartic acid in positions 232 and 97, respectively. This means that the higher observed enantioselectivity is due to a more subtle change in the 3D structure of this lipase.

**Fig 6:**
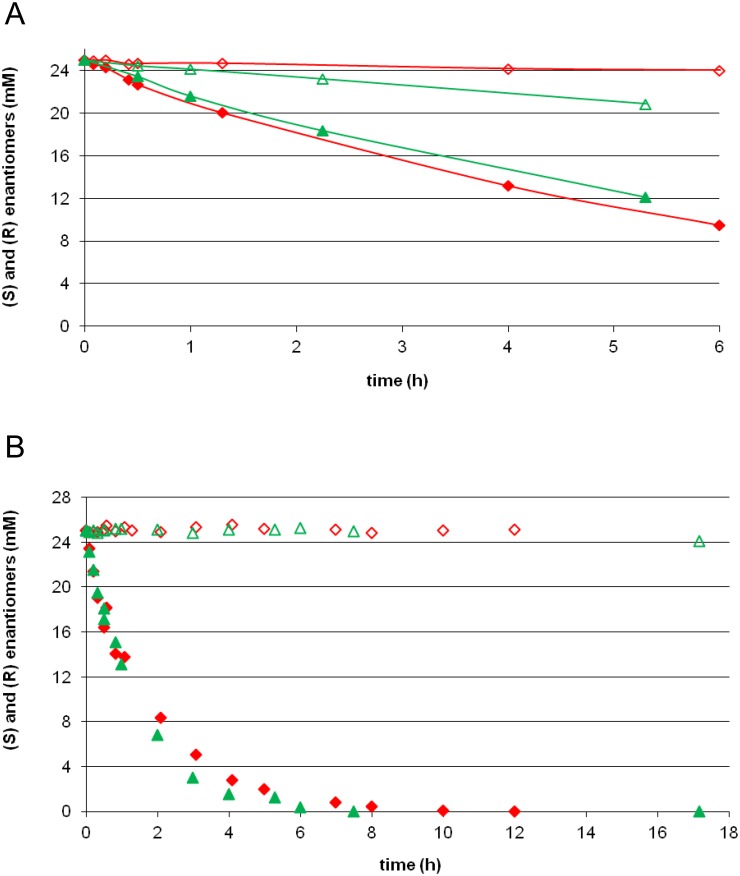
Hydrolysis kinetics of R,S-2-bromo-phenylacetic acid ethyl ester in a biphasic medium (water/decane v/v) at 25°C. Activities of YlLip2 (green triangle) and YpLip2a (red diamond), over-expressed in *Y*. *lipolytica* YJM1212, towards S- (filled symbol) and R- (open symbol) enantiomers. (A) Activities of the wild-type YlLip2 and YpLip2a. (B) Activities of V232S variant of YlLip2 and YpLip2a.

In a previous work, screening of a library of saturation of position 232 led to the identification of the V232S variant, with an E-value tremendously increased compared to the parental enzyme (E-value = 230) [18]. Valine at position 232 was changed by a serine inYpLip2a. After 12 hours of reaction, the preferred *S*-enantiomer was completely consumed whereas the concentration of the *R*-enantiomer remained unchanged (Fig 6). In addition to the gain in enantioselectivity, a 2.5 increase in velocity was observed (Table 5). With this variant, both enantiomers can be recovered with 100% purity and 100% yield. Finally, based on a previous work on YlLip2 [52], a double-substituted variant was constructed in YpLip2a by site-directed mutagenesis, the variant D97A-V232F. This latter presented reverse enantioselectivity with a total preference for the *R*-enantiomer (Table 5).

## Discussion

The objective of this work was to perform a comprehensive analysis of all lipase genes linked to YlLIP2 in a group of closely related species, with a strategy combining multiple approaches, including genomics, transcriptomics, structural analysis, enzymology and biochemistry. We wanted to test the idea that screening natural lipase diversity may help to maximize the success of the design of an optimal LIP2 enzyme. Indeed, from a general point of view, the chances of success are considerably increased when the selected enzyme naturally performs well in terms of activity and selectivity.

Our starting point was Lip2p lipase from *Yarrowia lipolytica* (Yllip2) which was shown to be an efficient catalyst for the resolution of 2-bromo-arylacetic acid esters, an important class of chemical intermediates in the pharmaceutical industry [15]. However, although its activity is satisfactory, its enantioselectivity still needs to be improved. Our strategy was thus to look for a better enzyme which could be produced by a member of the *Yarrowia* clade. The strength of our analysis was to successfully reconstruct the scenario for the evolution of the LIP family which gave rise to 16 lipases in YALI. The number of evolutionary events was not uniformly distributed among the different groups of lipases. The LIP2 family has undergone the highest number of evolutionary events, leading to identifying 11 lipases homologous to YlLip2 among the 59 putative lipases found in this study. In *Y*. *phangngensis*, five duplications of *LIP2* took place, which constitutes a reservoir for the detection of promising enzymes. One surprising finding of the high-throughput transcriptome analysis was the fact that none of the duplicated copies of *LIP2* was expressed in the conditions tested. From a fundamental point of view, this result deserves to be investigated at a larger scale, i.e. in all expanded protein families of these genomes.

Ten out of the 11 Lip2 lipases were cloned and expressed in *Y*. *lipolytica*, but only six were active using the classical *pNPB* hydrolysis test, whatever the target PrePro-peptide used for secretion. Three of the active lipases belong to *Y*. *phangngensis*, whereas only YpLip2a was found to be expressed in the wild type strain under the conditions tested. The fact that the enzymatic activity of the two non ancestral YpLip2b and YpLip2c has been conserved across evolution implies a selective pressure acting on these genes; otherwise this activity would have been lost, as in the case of YpLIP2d and YpLIP2e. The evolutionary scenario revealed that YpLIP2b and YpLIP2c derived one from the other due to a recent duplication. The alignment of the flanking regions of these two genes showed that the duplication event included both the promoter and terminator, which however are different from those of YpLIP2a. From an evolutionary point of view, it is interesting to note that the duplicated region exceeds the transcribed part of the gene, suggesting that the mechanism responsible for the duplication did not involve an RNA intermediate, but rather a segmental DNA duplication (S3 Fig). From this evolutionary analysis, we can deduce that YpLIP2b and YpLIP2c probably have the same expression regulation, if any, but the conditions of induction remain to be determined.

The Lip2 lipase from *Y*. *lipolytica* has been reported to be an efficient stereoselective enzyme for the resolution of 2-halogeno-arylacetic acid esters. In our study, the screening for enantioselectivity and activity led to the identification of the *Y*. *phangngensis* lipase Yplip2a which has a tremendously increased E-value compared to the first identified YlLip2 for the resolution of 2-bromo-phenylacetic acid ethyl ester (9-fold, E-value 30). In addition to the gain in enantioselectivity, a 2-fold increase in velocity was observed. Finally, the best candidate was subjected to enzyme engineering by site-directed mutagenesis targeted to the active site. The mono-substituted variant V232S showed significantly enhanced selectivity (E-value > 200) compared to wild-type enzyme. The improved E-value was the consequence of an increase in the reaction rate of the fast-reacting enantiomer, leading to a 2.5 increase in velocity. This variant performed better than the corresponding variant of YlLip2 in terms of enantioselectivity due to complete non-recognition of the non-preferred enantiomer [18]. A second double-substituted variant D97A-V232F of YpLip2a presented reversed enantioselectivity, with a total preference for the *R*-enantiomer, as already observed with YlLip2 [52]. The two YpLip2a variants are now compatible with industrial applications in the pharmaceutical industry.

This study is an interesting example of the power of using natural biodiversity exploited by comparative genomics for selection of promising enzymes in case of a multiple gene family, and for further enzyme property optimization. Facilitated by the advances in high-throughput sequencing technologies, this strategy should become widespread in the next few years.

## Supporting Information

S1 Fig Alignment of the PrePro regions of eight Lip2 lipases. (PDF)

S2 Fig SDS-PAGE analysis of secreted LIP2 lipases. (PDF)

S3 Fig Dotplot between YpLIP2b and YpLIP2c from *Y*. *phangngensis*, within a 3-kb DNA region. (PDF)

S1 File Amino acid sequences of the 59 full-length lipases of the *Yarrowia* clade and *C*. *hispaniensis*. (PDF)

S1 Table Characteristics of the 61 lipase genes of the *Yarrowia* clade and *C*. *hispaniensis*. (XLSX)

S2 Table Expression data of the lipase genes in YALI, YAGA and YAPH in three media (oleic acid, glucose and tributyrin). (XLSX)

S3 Table Primers used for lipase cloning. (DOCX)
