# Supplementary material for: Comprehensive Analysis of a Yeast Lipase Family in the Yarrowia Clade
Source: PLoS One. 2015 Nov 18;10(11):e0143096. doi: 10.1371/journal.pone.0143096 (PMC4651352; doi:10.1371/journal.pone.0143096)
Supplement: S1 Fig — (PDF) [file pone.0143096.s001.pdf]

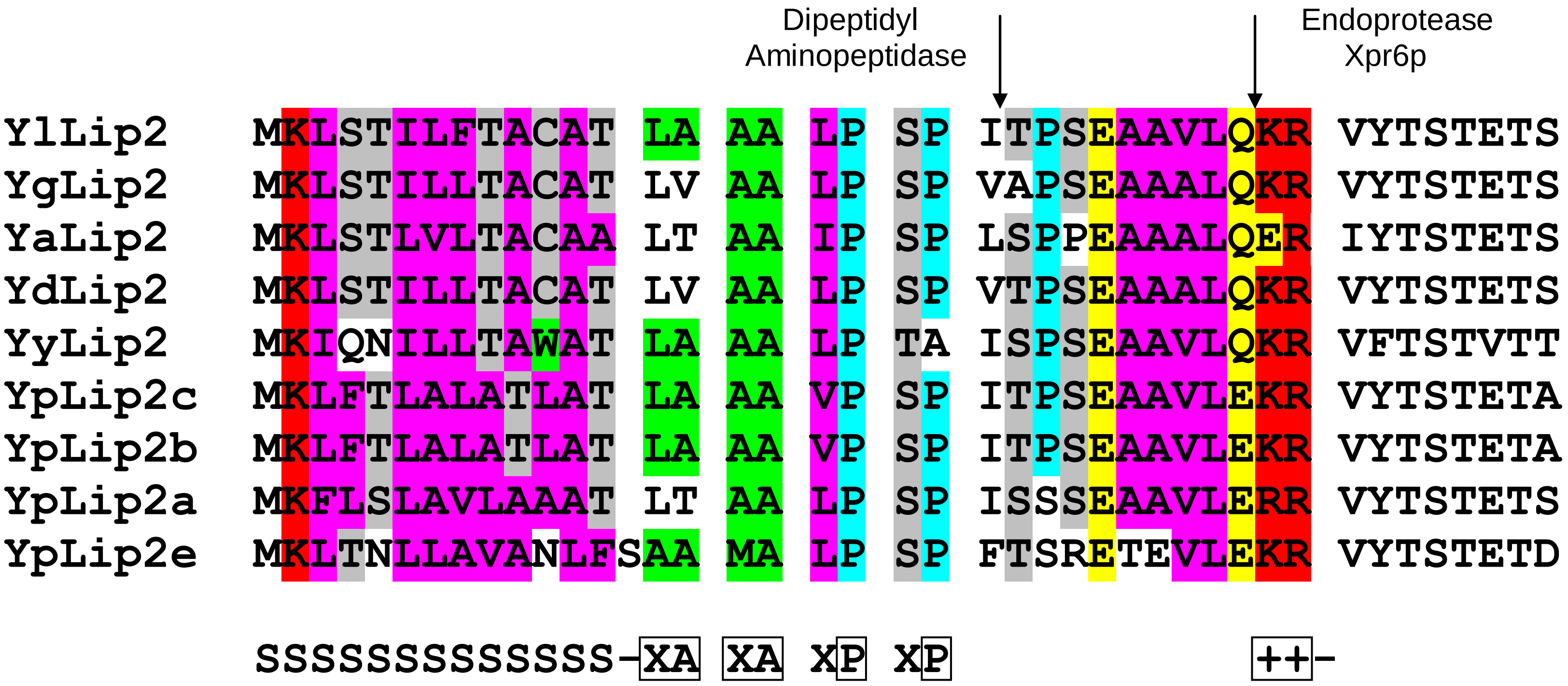

Additional Figure S1: Alignment of the PrePro regions of eight Lip2 lipases. Amino acids (aa) are colored according to their properties: positively charged aa in red, negatively charged aa in yellow, hydrophobic aa in pink, proline in cyan. Additionally, the X-Ala dipeptides are colored in green.
