# Supplementary material for: Comprehensive Analysis of a Yeast Lipase Family in the Yarrowia Clade
Source: PLoS One. 2015 Nov 18;10(11):e0143096. doi: 10.1371/journal.pone.0143096 (PMC4651352; doi:10.1371/journal.pone.0143096)
Supplement: S2 Fig — (PDF) [file pone.0143096.s002.pdf]

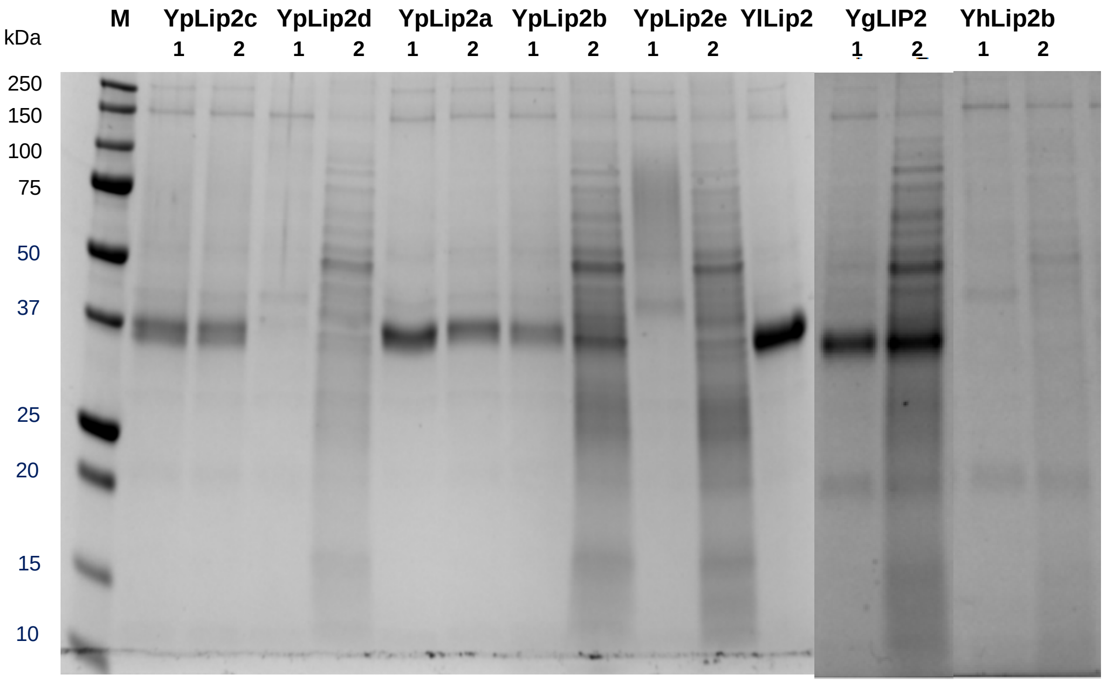


**Additional Figure S2: SDS-PAGE analysis of secreted LIP2 lipases.**

LIP2 genes were cloned in *Y. lipolytica* strain JMY1212 under the control of the constitutive TEF promoter from *Y. lipolytica*. Two strategies were adopted: either the entire genes were cloned with the presence of their own PrePro sequences (lines 1) or the part of the genes encoding the mature proteins were cloned behind the PrePro region of YlLip2 (lines 2).
