# Supplementary material for: Comprehensive Analysis of a Yeast Lipase Family in the Yarrowia Clade
Source: PLoS One. 2015 Nov 18;10(11):e0143096. doi: 10.1371/journal.pone.0143096 (PMC4651352; doi:10.1371/journal.pone.0143096)
Supplement: S3 Fig — (PDF) [file pone.0143096.s003.pdf]

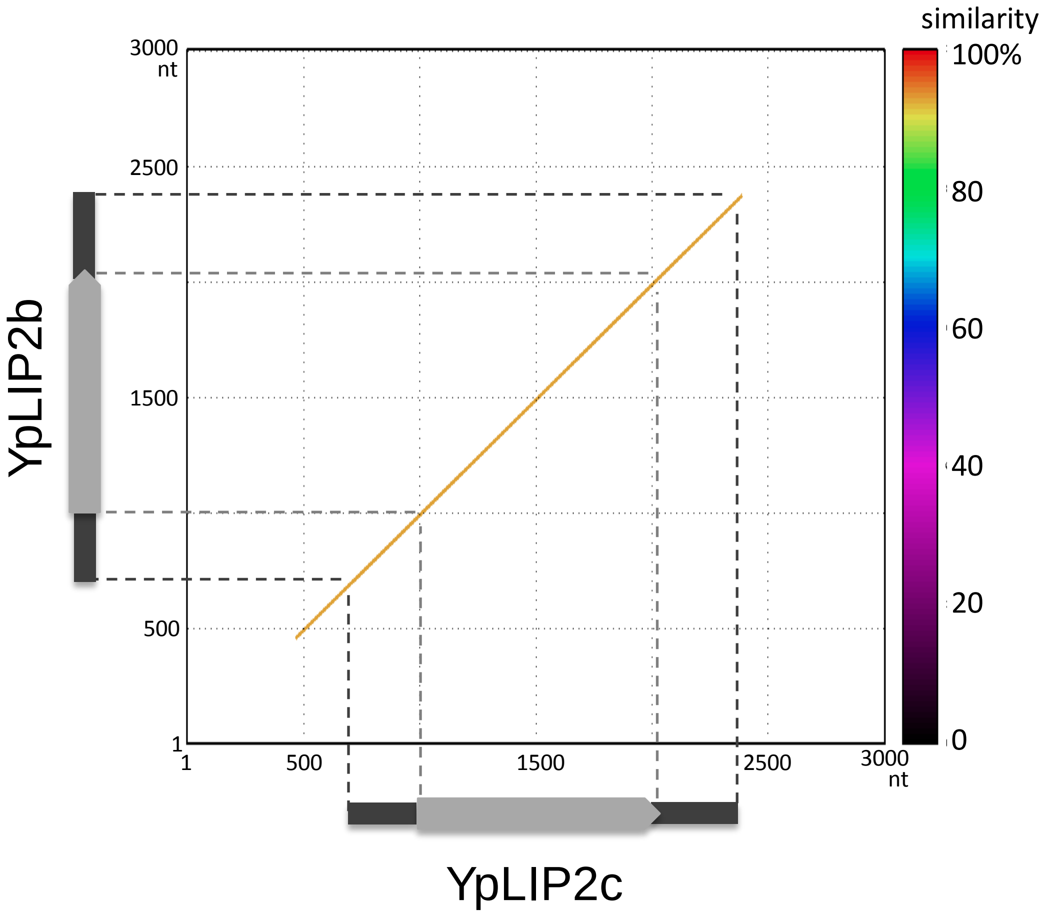

Additional Figure S3: Dotplot between YpLIP2b and YpLIP2c from *Y. phangngensis,* within a 3-kb DNA region (sizes are expressed in nucleotides, nt). The colour scale for similarity is given on the right. The CDS are depicted by grey arrows, the 5'- and 3'-UTR by dark grey rectangles. The coordinates of UTRs were deduced from the RNAseq reads obtained in the 6 samples (two replicates of 3 conditions: glucose, oleic acid and tributyrin). As shown by the orange line, the region of similarity exceeds the mRNA delimitation, suggesting that the duplication mechanism did probably not involve an RNA intermediate.
