# Supplementary material for: Comprehensive Analysis of a Yeast Lipase Family in the Yarrowia Clade
Source: PLoS One. 2015 Nov 18;10(11):e0143096. doi: 10.1371/journal.pone.0143096 (PMC4651352; doi:10.1371/journal.pone.0143096)
Supplement: S1 File — (PDF) [file pone.0143096.s004.pdf]

Additional File S1: Amino acid sequences of the 59 full-length lipases of the *Yarrowia* clade and *C. hispaniensis*

>YAYA0S1-32616g LIP10
MIFQTLLVFSALLGLSIADPNAVLLGDAPPQRTYSDRVIQATQQTWDWMNYALHLSGLAS
CVGWQGIEYPFSCDSYCGDYPGMELIAEFGPKDIIDFSVSGFFSVDHTRKEIWHVFRGTV
SLTDAVSDVRLDTQPFDAWGPNHPNCSDCQAHAGFLKSYDLAYQQFEKNMTDAFQKYPDY
SLKVTGHSMGGAAAYVHGISMKTRGYDPYVVASGQPLVGNQALADYNDKLFFGDKPDFLR
QDEDRRYWRLTHKGDLVPQIPFWSPFHQPGGEIYIDYALADPPLDSLKVCDGQDNPNCNY
SSSMVYAALGGTLLWAHFQYFVIFTLCGVNYWSTHIHDG
>YAGA0F31230g LIP10
MKLPTLLLITTFLGLSTADPNAVLWGSAPAQKTYSDRVVQATQETWDWMNYALHLSGLAS
CVGFQGIQYPFTCDSYCADYPGMELIAEFGPKDIVDFSVSGFFAVDHHRKEIWHVFRGTV
SLTDGISDVRLDSLPFDAWGPNHPNCTDCKAHAGFLQSYKLAYAQFEKNMTEAFQKYPDY
SLKVTGHSMGGAASFIHGINMKTSGYDPYVVASGQPLVGNQALANYNDMLFFGDKPDFLR
QDKDRRYWRLTHKGDLVPQIPFWSPFHQPGGEIFIDYALADPPLDALKVCDGQDNPNCNY
SSNMINSAITGTVMWAHFQYFVVFTLCGVNFWSTHIHS
>YALI0F11429g LIP10
MKLSTLLLYTTLLLVGFSAADPNAVLLGSAPPQKKYSDRVVQATQATWDWMNYALHLSGL
ASCVGFQGIQTPFTCESFCADYPGMELIAEFGPRDVVDFSVSGFFAVDHSRKEIWHIFRG
TVSLTDGISDVRLDTLPFDAWGPNHANCSDCQAHAGFLQSYNLAYAQFEKNMTDTFKKYP
DYSLKVTGHSMGGAASFIHGINMKTRGYDPYVVASGQPLVGNQALADYNDRLFFGDKPDF
LRQDSGRRYWRLTHKGDLVPQIPFWSPFQQPGGEIYIDYVLSDPPLDSLKVCDGQDNPNC
NYSSNMVNSAITGTLLWAHFQYFVVFTLCGVNYWSTHIHG
>YAYA0S1-12420g LIP11
MKLLSVFSTLTSLLSLTTAQNAVLPADQATWDLLNRVMYVSGNAVCVGQQGIQYPFTCDA
FCSSFPDFELITSWDNLQPLEFDVSGFLAVDHKRKEFWHVFRGTNTLKEQITNLQVQQQP
MAKWYSPELAQCDNCTAHQGFQIVYHNAYELFGDKMKETWAKYPDYKNVVTGHSLGAAAA
YLHGLNLKTSGKDPLVITSGQPLVGNRALAEFNDRLFFGDKPDFTALGPSRQFYRLTHRE
DIIPRLPFWDPYYHSGGEIFIDYPWSQPPLEDLKVCDGAENPNCVFSTSILEDGTVGIVE
LAHLIYFINFSLQCAIPLSPAYTGN
>YALI0D09064g LIP11
MKASSMCSNQINQLAGFILDLHLSIFVLLMLQYSSETFLNRVVEGWSGAKGPENPTCKVI
KRSTRSRRNSPESWTVSFSFFFASSFCSFSCFAMLLSSLVLSALSLVSVTAQNVVQATQD
TWDLINYAEHLSSLAICGEPYGVIYKPFQCAGRCSDFPDMELITQFTPQDPLDFSVSGFL
AVDHKRKVFWHVFRGTATLNNGLTDLRIKRQPLTSWNTAKMDCPDCQVHVGFLQAYNLAY
SEAKGAMDDTFAKYPDYQVIVTGHSLGGAATFLHGINLKTSGYDPLVITSGQPLTGNKAL
ADYNDKLFFGDNPDFTHQGPDRRFYRVTHKEDIVPRIPFWTPYHQSGGEVYIDFPGINPP
VNTLKVCDGQQNPLCSFSTSLASTATQGIVEAAHLIYFTFFFLCSTLLYPPLNSDLPVGV
WGKPLNGTI
>YALI0D15906g LIP12
MRPSITIVGFLIASAVALGITQASKDTYNLLNYAENLNGVAVCVEQIGGIQKPFKCLGHC
DDFPDMELITTFHPKKLFDFSTTGFLAIDHKRKQFWHVFRGTASLTDGISNLRLERQPLV
FWDNPEFDCPGCEAHEGFLTAYNDAYDQIRDVLNQTLAQYPDYQIIVTGHSFGGASSFLH
GINLKSQGMDPLVITSGQPLTGNKALADFNDKLFFGDNPDFTYQGPDRRFYRVTHKDDLV
PRLPFWNPFHHSGGEVYIDYPLTNPPLRTLKICDGQQNPRCSFSTSLITAALLGTLQQAH
FMYFTFFSLVCGVNIAGHLGPPLQ
>YAYA0S2-00584g LIP13
MRLLTAIGFLLLLITQTQAISRATYELLQFHSQLSDIAYCVQTVRGPTQLRRPFKCGVQC
RKAQLEGVEVVHTFVHSPASPALTGYMAVDHTNQTKYVVFRGTNSLEDSMLDLSFGHEPT
ASPSFLKSTCPECKVQGAIMQAYDAFWAENSIQLSDFLYENFSQYSLSVTGHSLGGVAAA
LLATDLKLQGLDPLLINFGQPQYANLAYAQLVDSLFFPPADKEVIDPLYDSPQRRLYRVT
HWNDVFVTLPGQRGFFHSLGEVYISYPTVNPPRRTIRYCEGPESEYCHSGDYNPFERANF
LKNHLAYFGWVGYCPYL
>YAPH0S2-22078g LIP13
MILASTTIFLWIFQLILAQTVSQQTYDLLKFHARLSDIAYCVQPVNSPTQLRQPFECGVK
CTNIEGVELFHSFVHKPGNTGFTGYFAFDHVNKTKYIVFRGTNSFEDTLVDFSMSHELPC
ETPFFLENLCPECKVQSSLLEVYGLFLEQHVTPLWDFVYTFYPDYSISVTGHSLGGVAAA
FLATHLRVQGLEPVLVTFGQPRYGNLAHAEFIEKIFLTSQRRLYRVTHWNDVFVSLPSSE
QFSHFSGEIYISHPWVDPPLETVRYCEGRESETCHAGDYNPVERVTFLKNHLAYFIWVGY
CPYR
>YAAL0S01-08218g LIP13
MWITVLPILFFLPALVLAISAPTYDLLAWHSQLSDVAYCVQTVRGPTQLRTPFKCGKQCR
EPQMQDMQLIHAFVHSPARPALTGYLAVDHASHTKYVVFRGTNSIEDTVLDLSFGQQPSV

SPGFLQGACPECQVQGGILAAYETLWEENSGPLSDFLHSQYPNYTLSVTGHSLGGVAAAL
LATHAKLEGLDPTLVTFGQPRYANLAYAQLVDSLFFPLNGSDGGSNLGPLDESPRRRMFR
VTHWNDVFVSQPELGGFHHSLGEVYISYPWVSPPRRTVRYCDGAQSEYCHRGDYNPLERA
NFLKNHLAYFGWIGYCPYL
>YAGA0E34992g LIP13
MLLRILPTTLVLLFFLVTQTHAISEATYELLAFHSQLSDIAYCVQTVRSPTQLRHPFKCG
VQCRKTQFEGLEVIHTFVHKPASPALTGYMAVDHTNQTKYVVFRGTNSLEDSMLDLSFGY
EPTASPSFLKDACPECKVQGAILEAYEAFWAENCDQLSEFLYEDYPHYSLSLTGHSLGGV
AAALLATDIKVQGLDPLLINFGQPQYANLAYAQLVDSLFFPQTDKEVIDPLYDSPQRRLY
RVTHWNDLFVSLPGQRGFFHSLGEVYISYPWVKPPRRTIRYCEGPQSEYCHGGDYNPLER
ANFLKNHLAYFGWIGYCPYL
>YALI0E00286g LIP13
MRPFTTLVLLLLLITQTCAISEATYELLQFHSQLSDIAYCVQTMRGPTQLRHPFKCGVQC
RKTQFEGLEVVHTFVHKPARPALTGYMAVDHTNQTKYVVFRGTNSLEDSMLDLSFGYEPT
ASPSFLNDTCPECKVQRAILEAYDAFWAENCDQLSDFLYEDYPHYSLSLTGHSLGGVAAA
LLATDIKLQGLDPLLINFGQPQYANLAYAQLVDSLFFPQTDKEVIDPLYDSPQRRLYRVT
HWNDLFVSLPGQRGFFHSLGEVYISYPWVKPPRRTIRYCEGPQSEYCHGGDYNPLERANF
LKNHLAYFGWIGYCPYL
>YAGA0F20142g LIP14
MVLASSTVFAEWFFRVLLGTVAPSPQTATAPVTQEFYDTALTYSHLSNIAYCINAPFESL
KTDFSCGVACSHFPNMELVEAFGGEFFETSITGYLAIDHVKKEKYVVYRGTFDIGDVYTD
IQLAQSPYLVTASMFDSPDNLCEGCTIHDGFNKAYKETMVNIGDKLEQHLTNNSDYKLVV
AGHSLGAAIAVLSATSIKAKGFDPYLFTYGQPRIGNANFAQFVSKMWFGDGDGLSMGPDR
RLFRISHWNDLFVGFPAFKDYVHSVGEIYIDYYTVNPPLNTLYSCAGPESMSCYRKDFNA
IERANILKNHLAYIDWLSLCTLNVGRRELQERGRRFEGKFLYGGIANGTIIF
>YALI0B11858g LIP14
MVLSTVIGEWFSRVLFGTVAPSPLTATAPISQDFYDTALTFSHLSNVAYCINTPLESLKS
DFSCGVACSHFPNMELVEVFGGEFFETSITGFLSIDHVKKEKYVVYRGTYDIGDVYTDIQ
LSQSPFLVTPSALGSTANLCEGCTIHDGWNKAYNETMGIIGDKLADHVNSNPDYRLVVTG
HSLGAAIAVLSATSLKVNGQDPYLYTYGQPRIGNANFANFVSKQWFGEGDGLSMDSDRRY
FRLTHWNDLFVGFPAFKDYVHSVGEIYIDYFTVQPPLNKVFSCAGPESMSCYRKDFNALA
RLDIVKNHLAYFDWISLCTLNIGRRDLERGRKFEGTWLYGGLANGSTIF
>YAPH0S3-09582g LIP15
MVSWYTQFKAAILSLLGMTASTPSTTIASISQGMYNEISYFSRMVNTAYCANAPITPLRT
DFSCGDSCQYFANLKLDSIFGGNFYSTSATGLLAHDHKRKEKYVLFRGTFSIPDAYTDIQ
FQKSPWLAKLPNGVVPKIQSAGGQPLTCEGCAVHDGFAKAFNETLKNSGHQFDKFLANHT
DYKLYVVGHSLGGAMAQMFAVRLKLMGYDPTLITYGQPRVGNKEYAEFVSRLFFNDESGL
LVDENRRLYRVTHWNDIVVGLPNFADYTHSVGEVFIASEDLNPPVESVTLCEGAENEACH
RGHFSLWERVKILHNHLAYINYIGYCALNIGRRSILNMPNYRGKNTYAHKSETTKDE
>YAAL0S01-09208g LIP15
MQFTFGQVMALLYSTLFGRPPASKETTRVYVDQSMYDFTAKFSRLCNVAYCVDAPITPLR
TDFSCGESCRYFPNMTLDAVFGGDFYSTSITGYTGTDHHLKEKYIVFRGTFSIPDIVTDI
QFQRSPWLVQLPPIVTTKYNDFKPAARANCTDCQIHDGFAKAFNETLQNAGPQINDFLSN
NTDYKLYVAGHSLGAAQAQLFATSFKLQGYDPILVSYGQPRIGNKEFADFVSELFFDGDD
GLSMNDTRRMYRFTHWNDIFVGLPDWGNYTHSVGEIYIDQRDVYPPLNAVTACAGPENPD
CHRGTFNLWEQINLFQNHLAYIYYIGYCALNIGKRDVFNMPKYTGNYTYGHRSEYQTNLP
>YAGA0E29558g LIP15
MNLTLGQVVAYLYASLFGEPPATLSKTKVQASQDLYNFTAKFSRLANIAYCVNAPVTPLR
TDFTCGESCRYFPNMTLDSVFGGDFYSTSITGYIAYNHAEKEKYVVIRGTFSIPDAVTDI
QFQNAPWLVQLPEHLIPTQTDMQQKWAVRPDLEVENKGLDSLKERTPLTEDPRLIPIKTK
ECKGCMIHDGFAKSFNETMANAGPQFEKFLTNHTDYKMFITGHSLGAAQALLFGTHFKLL
GHDPTMINFGQPRVGNSEFANFINQLWFNDTGLIVDDKRRNYRITHWNDIVVGVPDWMNY
THSIGEVYIDYEDVNPPLNKVAVCEGGENEACHRGTFNLWSRINLLQNHLAYIYYIGYCA
LNIGRRDVMAMPKYHGNFSYRYGSDPGYSGNGRTELPTTN
>YALI0E11561g LIP15
MTFTIGIMNLTLGQVVAYLYASLFGEPPATLSKTRVQASQELYNFTAKFSRIANIAYCVN
APVTPLRTDFTCGESCRYFPNMTLDSVFGGDFYSTSITGYIAYDHLNKEKYVVIRGTFSI
PDAVTDIQFQQSPWLVELPKHLIPTQDDMKQRWAVRPDLEVENKGLDNLKERALVVEDPR
LIPIKTSECKGCMIHDGFAKAFNETMVNAAPQFEKFLTNHTDYKMYVTGHSLGAAQALLF
ATHFKLLGHDPTMINFGQPRVGNSEFANYINQLWFNDTGLEVNDKRRNYRLTHWNDIFVG
LPDWSNYTHAIGEVYIDQESVYPALDKVSVCEGGENEACHRGTFNLWSRIDLLQNHLAYI
YYIGYCALNIGRRNVLAMPKYHGNNSYKYATEPGYTGPGRTLVPTN
>YAAL0S06-02102g LIP16
MKLIAFILVLAGMLSAVHAQTKAITQDTYDLILKYGQLCNVAYCVRAPGPFGLQDNFTCG

KACSHFPDTELVYKFGGNFFSTSITGFLAVNHEKKEKYIVFRGTFSIADAITDIQFLQKP
YLADLPPLNTTNINSTHPLARPECPGCEVFDGFQKAYRETMVNMGDNLINHINSNPDYKL
IVTGHSLGAATGLLMAINLKNLGLDPVVVLYGQPRVGNKAFADYANSLFFDKGSNGVDIT
NSTRLYRVTHWNDVVVGLPFWSGYTHTIGEVYISYPNVASPIRYVHSCAGASNRACHSGN
FNLFARFNILKNHCSYLSWIFYCAINVGKRDLIHDPPRVSSGMKHWSEGQFSDQSEQQIY
DAVFPN
>YAGA0D12134g LIP16 [intron]
MLSLITLFLFIAQAYAQIANITQPTYDFILKYGQLSNVAYCVRALGPFDLTDQFTCGKSC
AHFPNMTLDYQFGGSFFSTSITGFLAHDHTKKEKYIVFRGTFSLADAYTDALFFQEPYMA
DLPPLNTTNINSTSTEARADCPGCEIHDGFQVAYRETMENMQDHLVDFLKNNTDYKLIVT
GHSLGAATSLLMAINLKNLGFDPMVITFGQPRVGNKAFADYANSLFFKEGDNGMDINPER
RLYRVTHWNDIVVGVPFWSGYTHTLGEVYISYPDVGSPIEYVNACAGPDNSQCHYGTFDL
LARVNILKNHCSYLNWIFYCAFNVDKRDMMIDPPRIDKRVQHWSGKFADVEVGERMMYEA
SYPM
>YALI0D18480g LIP16 [intron]
MLSLIAIFLLVTTALAQTANITQSTYDFVLKYGRLSNVAYCVKAPGPYELETDFTCGRSC
GHFPNVTLEHQFGGDFFSTSITGFLAHDHTKKEKYIVFRGTFSLADAITDALFLQEPYLA
DLPPLNTTNINSTSNSARVDCPDCEIHDGFQKAYRETMVNMQGHLVAFLRNNTDYKLIVT
GHSLGAATALLMGINLKNLGFDPMVITFGQPRVGNKAFADYADSLFFKQGDNGLNINPER
RLYRVTHWNDIVVGVPFWSGYTHTLGEVYISYPDGVNAPIEYVNACAGPDNDQCHYGSFD
LLARVNILKNHCAYLNWIFYCAFNVDKRQMMIDPPRIHKRVEHWSGKFADVEFSERMIYE
ATYPM
>YAGA0F44122g LIP17 [intron]
MLSFITIFLLATQALAQTAAISQSTYDFVLKYGWLSNVAYCVRAPGPFALQDNFTCGKSC
AHFPNVTLDYQFGGNFFSTSVTGFLAHDHTKKEKYIVFRGTFSLADAITDIQTVQQPYMS
TPPPLNTTNINSTDPSANIDCPGCEVHDGFQKAYRETMTNVQDHLVEFLGKNKDYKLIVT
GHSLGAVTALFMGINLKNLGYDPTMINYGQPRLGNKAFADYVDALFFKKGDDGLAITPER
RMYRVTHWNDFFVGWPAGYTHTIGEVYISDPTGINAPLQDVYACAGPENDKCHHGSFNIL
ERLNILKNHCAYLNWIFYCAINVDKRQMMIDPPRWTAGNSDGVELTERMMYEATYPM
>YALI0F32131g LIP17 [intron]
MLSFIALFLLVAQALAQTAPITQETYDFVLKYGWLSNVAYCVRAPGPFALQSDFTCGNSC
AHFPDVTLDYQFGGNFFSTSVTGFLAHDHTKKEKYIVFRGTFSIADAITDIQTIQQPYMT
SIPPLNTTDINSTNPSASINCPGCQVHDGFQKAYRETMVNVQDRLVDFLGNNTDYKLIVT
GHSLGAVTALFMGINLKNLGYDPTLINYGQPRLGNKAFADYVDALFFKQGDDGLTINPER
RMYRVTHWNDFFVGWPAGYSHTLGEVYISDPTGINAPIEDVYSCAGPENNQCHHGSFNLL
ERLNILKNHCGYLNWIFYCAINVDKREMMIDPPRVGKRVEHWSGKFSDVESTEGLMYEAI
YPM
>YALI0B20350g LIP18
MIVLLLLLSVALSSPIMLDRFGDSVNRMIRGNSEAYESGETRGVSQEFEQRLVRYMWFNN
VAPCVPKKLQHPFKCIARGCKELGKHTELVDIFTHSDNLFDRTISGFVALDHKHKEIVLA
LRGTQDAHDWVTDLHLRLVGLHPEHLGVSNFNCRNCQVDLGFLKGYLHSFHVVDSIVQRL
TEKYPDYQLVITGHSLGGTAATLFGLNYRLNGYSPLVFSAGAPALGNKQFANFADRVFWG
SQNPNTLKVKERDIKFCRMTHLGDFVPRFPFWNGYQQMSGEVFINDVRGIYPPRETLQRC
NGQQNRQCSFGDQYRKLEMNFKPHSAYLVPGSECSLSGRRELTFGQVHAANDTNKDSDTN
IEPDIEPILIPVVQID
>YAYA0S7-05842g LIP19
MILLFLFLCFALSSPIGMDRFSDGVNRMIRGNAGAFESSETLGVSREFEQRLIRYMWFNN
VAPCVPKKLQHPFKCIARGCKELGRQTELVDIFTHSRNLFDRTISGFVALDHKHKEILLV
LRGTQDINDWVTDLHLRLVDLQPEHLGLTNLNCRNCQVDQGFLKGYLHSFHAVDSIVRRL
IEKYPKYQLVITGHSLGGTAATLFGLHYQLHGSSPQVFSAGAPVLGNKQFANFADRVFWG
SQNPNTLIVKENDIRFCRITHLGDFVPRFPFWKGYQQMSGEIFINDVRGIEPPRDALKRC
NGQQNDKCSSGDQYRHLEMNFKPHSAYLVPGSECSFSGKGELTLGQIQAAKNTYQDTDTD
TDIEAVLIPVVQLD
>YAPH0S5-11738g LIP19
MLIIFLLTLIASASPFIRLALGEKLNQLLVGSHEAFESEETQLISPEFQECLIRYMWFNN
VAPCVPKQLEHPFVCAARGCKMLGKYTELVDIFTHSESRMDMLTDRTITGFVALDHLHKE
IVLVLRGTQDVNDWLTNLQVSLVGLNPADLGVKSLNCPNCRVGLGVLSGYISSQRVADPI
IRRLKEKFPQYQLVVTGHSLGGTAATLFGLNYQLNGLSPKVFSVGAPALGNKEFTNFVDE
VFWGSEHPDTLSVPEKNINFTRVTHLGDFIPRFPFWKGYQQMSGELFIHDVKGIDPPLDK
LRRCNGQQNRKCSFGDKFRQLTLNIRSHSAYLVPRNRCSGERRKIMGDIGAYGNDTVLIP
VVDLDSLE
>YAAL0S01-04962g LIP19
MILFFFLLICVGLSSPLLNRVGTGVNRMIGGSDEAYQSHETVAVSSGLEKRMTRYMWYNT
VAPCVPKRLQHPFKCEAQGCTKVGTQTELVDVFTHSGDVLDVFTDRTISGFLALDHKNKE

ILLVLRGTQDANDWVTDLRLRLVPLEASHLDAPSIGCHGCQVNVGFLEAYQHTWRVVDST
IRDLKKRYPNYQLVLTGHSLGGTAAILFGLNYKLNGLHPTVFTAGAPAIGNKQFANFADQ
VFWGSSNPDTLSVPENRLCFLRLTHRGDLIPRFPFWGGYQQMSGEIFINDIRGIYPPLSS
LRRCNGQQNNRCSFGDQNRQLEMNFRPHSAYLVPGSECSFSNREGHIGRGGAGDMVNAMD
TANTTEDTTDTTEMDPGLLAVEIPVVAGPV
>YAGA0F28348g LIP19
MIVLFLFLCVALSSPIVLDRFGDGVNRMIRGNSEAFESGETRGVSRYFEQRLIRYMWFNN
VAPCVPKKLEHPFKCIARGCKELGKHTELVDIFTHSDTLFDRTISGFVALDHKHKELVLV
LRGTQDANDWVTDLHLRLVELRPEHLGVSSFSCRNCQVDLGFLKGYLHSFHAVDSMVRRL
TELYPKYRLVITGHSLGGTAATLFGLNYQLHGFSPLVFTAGAPALGNRHFSNFADRVFWG
SQNPNTLKVKEGDIKFCRMTHLGDFVPRFPFWSGYQQMSGEVFINDVRGIDPPRESLQRC
NGQQNKKCSFGDQYRQLEMNFKPHSAYLVPGSECSLSGRRELTVGQAESEVQAAAATNNT
DTYADTDFEPVLISVLQID
>YALI0A10439g LIP19
MIILLLFLSVALSSPIMLDRFGDSMNSMIRGNNEAYESGETCGVSQEFEQRLVRYMWFNN
VAPCVPEKLQHPFKCIARGCKELGKHTELVDIFTHSDNLFDRTISGFVALDHKHKEIILA
LRGTQDVNDWVTDLHLRLVELHPEHLGVSNFNCRNCQIDLGFLKGYLHSFPAVDSIVQRL
TEKYPNYQLVITGHSLGGTAATLFGLNYRLNGYSPLVFSTGAPALGNKQFANFADRVFWG
SQNPNTLKVKERDIKFCRMTHLGDFVPRFPFWNGYQQMSGEVFINDVRGIYPPRETLQRC
NGQQNRQCSFGDQYRKLEMNFKPHSAYLVPGSKCSLSGGRELTFGEVHSAANDTNKDSDT
DIEPDIEPILIPVVQID
>OLHI0B09560g LIP2a
MKFLTVLSLMACTAFAAPIDEVTTTPESLSFAVLSLFGSPSVGYESIDTAPVSQDTYNKL
LKYSRLAGISYCVGFETTIDKPFRCGLQCKEFPNTEVLYVYQDIQFDPTLPSYIAVDHNS
KEIYTVFRGTRSVGDAITDLEINQKSLTNFAWGKNITASQTCENCKVHEGFLRAYNLTYN
RISKNLDDVILKYPDYSQTIVGHSLGGAVALLFGISMKVNGHDPLVVTYGQPLVGNKEFA
DWADQLFFGQTKPNVLSDFPSRKFYRVTHLGDIVPTVPFWSGYTHTSGEIFLDQKNGVLP
SLKNVKFCQGQVNKKCAAGTPFNKKLILLEHLEYFILLGLCRI
>OLHI0C13190g LIP2b
MKFSALLASLAIGTTLARPLQEVEATNESGKNSQLLEARGYASVETAQVSQDTYNKLLKY
SRLTAISYCVGHITEVLKPFKCISYCSFFPHTELIKSYRDEFNDLSLTSYLAVDHDSKEI
YTVLRGSHSVEDWMVDFTVRQESIDNMVWGKNATASQSCQKCKVHKGFLTAYTLTYEKMH
NDIEATIAEYPEYSLTITGHSLGAAVALLLGVSMKVNGHNPLVVTYGQPLVGNQEFANWV
DTLFFGQTEPNALIDSPERKLYRVTHRGDVVTNVPSWGDYTHASGEVFLDTPVIMPKTSQ
VKFCQGQNNKKCSDGNLISQKLVMANHAHYFVSQANCLAL
>YAYA0S6-01420g LIP2
MKIQNILLTAWATLAAALPTAISPSEAAVLQKRVFTSTVTTPIDQDDYNFFQKYARLANI
GYCVGPLTQIFPPFTCGLQCAYFPNVELIQEFDDPLLLFDVSGYLAVDHNSQQIYLVIRG
THSLEDVITDLRVTQAPLTNFDLAANISATATCEDCLVHSGFMQSYNDTFNLIGPKLDSV
IAQYPNYQIAVTGHSLGGAAALLFGINLKVNGHDPLVVTLGQPIVGNAGFANWVDTLFFG
QENPDVSKVTPDRKLYRITHQGDIVPQIPFWAGYQHCSGEVFIDWPLILPPLSTVVMCEG
QSNSQCSAGNTLLQQANVLGNHLQYFVTQGICGI
>YAPH0S1-03994g LIP2c
MKLFTLALATLATLAAAVPSPITPSEAAVLEKRVYTSTETAEISEDSYHFLEKYSRLANI
GYCVGPLANIYKPFKCGLQCGKFPNAELIQEFHDPSYVFDVSGYLAVDHGSKEIYLVFRG
THSLEDAITDLRVQQAALTHFDNAANISSHATCDNCLVHNGFIETYNNTHKQVQAQLDAV
IKKYPDYKIAVTGHSLGGAAALLFGISLKLNGLDPLVITYGQPIVGNKAFANWVDKLFFG
KENPDVSKITPERKLYRVTHRGDIVTQIPFWDGYQHNSGEVYIGWPLISPPFSKVVTCQG
QSNKNCSAGNSLLSQLNVLANHLQYFVLMGICGI
>YAPH0S1-31032g LIP2d
MNFFKIFLLSAFAAALSIRQFKSTETYHIDQNAYDFFLKFARLTNIAYCVGPLTAIFKPF
TCGLQCAHFPNMELIRTFRDPAIVFDASGYLAVDHGAKEIYLVTRGTHSIEDVLTDFQVI
QAPLTNMDLAVNITAGDTCKDCRVHSGFLRAYNYTYNQISDTLNSVIKKYPDYKMVVAGH
SFGGATALLFAVNLKVNGHDPLLVTLAQPIVGNRAFANWVDRLFFGQENPDVSKFSPDRN
LYRVTHKGDLVAQVPFWAGYQHSSGEVFIDWPLVHPPLSKVVMCQGQSNSKCSAGNRLRD
QIHLVSSHLQYFVLQGICGL
>YAPH0S5-13278g LIP2e
MKLTNLLAVANLFSAAMALPSPFTSRETEVLEKRVYTSTETDPVTQNTYNLLEKFSRIAS
ISYCVRPPLKIMKPFKCGIPCEYFPDVELLQQFHEPEPFVFDVSGYVAVDHGSKQIYVVA
RGTKGLEDTITDLRVMQAPFDHFDLGTNNASVTADCKDCLVHKGFIQAYNNTMKQIQPVL
DSAVNKYPDYKVAVTGHSLGGATALLLGINLKVNGYDPLVITYGQPLVGNAGFANWVDKL
FFGQENPDTTQITADRKFFRVTHKGDIVTQVPFWDGYTHNSGEVFIDWHTLNPPLHKVVA
CKGQSNKNCSAGNKLVQQVYLKNHVLYFIYQGKCNAFNTFSD
>YAPH0S6-00584g LIP2a

MKFLSLAVLAAATLTAALPSPISSSEAAVLERRVYTSTETSQISQDSYNFFEKYSRLANI
AYCVGPLSNINKPFTCGLQCAKFPDVELIQEFRDPALIFDVSGYLAVDHGSKQIYLVVRG
THSLEDVITDLRIKQAPFTPFDMAANISSTDTCDNCLVHNGFIESYNNTYKQIGSKVDAV
IKQYPDYEIVVSGHSLGGAAALLFGINLKVNGHDPLVVTYGQPIVGNAGFANWVDRLFFG
QENPDVSQVTPERRLYRVTHRGDIVPQIPFWDGYQHCSGEVFIDWPLVNPPLSKVVTCQG
QSNKKCSAGNTLLQQLNVLANHLQYFVLQGICGI
>YAPH0S6-03488g LIP2b
MKLFTLALATLATLAAAVPSPITPSEAAVLEKRVYTSTETAEVSEDSYYFLEKYSRLANI
GYCVGPLANIYKPFKCGLQCGKFPSAELIQEFHDPDYVFDVSGYLAVDHGSKEIYLVFRG
THSLEDVITDLRISQAPLTHFDNAANISSHATCDNCLVHNGFIETYNNTHKQVRAQLDAV
VKKYPDYKIAVTGHSLGGAAALLFGISLKLNGLDPLVITYGQPIVGNKAFANWVDKLFFG
QENPDVSKITPERKLYRVTHRGDIVTQIPFWDGYQHNSGEVYIGWPLISPPFSKVVTCQG
QSNKNCSAGNSLLSQLNVLANHLQYFVLMGICGI
>YAAL0S04-06656g LIP18b
MKTFALLGLLASSVATAAATNASYADHYADVHFSNALGEVRPAPPPAAIIDYARGPQDTA
PITADFYNELEYYMNFSQVAACVGYAGIKKPFNCKVEGCKILQGHVELVSMLSPPELQQF
NVSGYIALDHLKKNIVLVTRGTLDIRDILTDVNILYRKMSPVDFAPNLQSENLKCDGCKI
HDGFLHSYLTTKEFVDPIVAQLKSQHPEYGMVVTGHSLGGATALLFGLDYKINGYDPLVI
TSGQPYVGNQAFADYHDTVWFGKPNPDTVTVGGDRKFYRLTHLHDIVPRVPYWGGYMQVS
GEIFIDYHKTHPPVDKVLACAGQLNNHCTHGSPWWTLFNLLDHFWYIEPFVPCF
>YAAL0S10-01222g LIP2
MKLSTLVLTACAALTAAIPSPLSPPEAAALQERIYTSTETSHIDQNAYNFFEKYARLANI
AYCVGPGTKIFKPFKCGLQCAHFPNVELIQEFHDPAFIFDVSGYLAVDHTSKQIYLVIRG
THSLEDVITDIRIFQQPLTNFDLAANISATATCSDCLVHKGFISSYNNTYNQIGERLDTV
IKQYPDYQIAVTGHSLGGAAALLFGINLKVNGHDPLVVTLGQPIVGNTGFANWVDRLFFG
QENPDVSKVTTDRKLYRITHRGDIVPQVPFWDGYQHCSGEIFIDWPLIHPPLSNVVACEG
QRNKQCSAGNLLIQQANVIGNHLQYFVTEGVCGI
>YAGA0E38754g LIP2
MKLSTILLTACATLVAALPSPVAPSEAAALQKRVYTSTETSHIDQDSYNFFEKYARLANI
GYCVGPGTRIFKPFNCGLQCAYFPNVELIQEFHDPLLVFDVSGYLAVDHASKQIYLVIRG
THSIEDIITDIRIMQAPLTNFDVAANISSTATCDDCLVHNGFIQAYNNTFNQIGPKLDSV
MTQYPDYQVAVTGHSLGGAAALLFGINLKLNGHDPLVVTLGQPIVGNEGFANWVDKLFFG
QENPDVSKVSKDRKLYRITHRGDIVPQIPFWDGYQHCSGEVFIDWPLIHPPLSSVVMCQG
QSNKQCSAGNTLLQQANVIGGHLQYFVTEGVCGI
>YALI0A20350g LIP2
MKLSTILFTACATLAAALPSPITPSEAAVLQKRVYTSTETSHIDQESYNFFEKYARLANI
GYCVGPGTKIFKPFNCGLQCAHFPNVELIEEFHDPRLIFDVSGYLAVDHASKQIYLVIRG
THSLEDVITDIRIMQAPLTNFDLAANISSTATCDDCLVHNGFIQSYNNTYNQIGPKLDSV
IEQYPDYQIAVTGHSLGGAAALLFGINLKVNGHDPLVVTLGQPIVGNAGFANWVDKLFFG
QENPDVSKVSKDRKLYRITHRGDIVPQVPFWDGYQHCSGEVFIDWPLIHPPLSNVVMCQG
QSNKQCSAGNTLLQQVNVIGNHLQYFVTEGVCGI
>YAYA0S2-08614g LIP4
MPAYDLTLGQVLAYLASMFSGQVDATSSSTRIQATQELYNFTAKFSRLANIAYCVDAPLT
PLRTDFTCGESCRYFPNITLDSVIGGDFYSTSITGYIAYDHAKKEKYLVFRGTFSIPDAV
TDIQFQNAPWLTSLPTNLIPNQNDKQTIGKEYVAENKGLGGLEERQAIVHEDPSLVPNKT
ATCDNCQIHSGFADAFNETLRNAGSNFDRFLRNNTDYTMYVLGHSLGAAQAQLFATRFKL
LGYDPHLINLGQPRVGNAEFAAYINQLWFNDTGLIVNDARRLYRLTHWNDVVVGVPDWLN
YTHSIGEVYIDVENVYPTLDKMVVCEGGENPACHRGTFNLWARIDLLQNHLAYIYYIGYC
ALNIGRRDILNLPKYHGNVSYQHGSDPNYNYDTKAPTKLK
>YAGA0E25950g LIP4
MAGFNFTFGQVMSYLLSMFYGQVDATSSSTRIQATQDLYDFTAKFSRLANIAYCINTPFV
PLRTDFTCGESCRYFPDLQLDSVFGGNFSSASTTGYIAYDHKKKEKYIVFRGTFSIPDIV
TDIQFQTAPWLASLPNHLIPTKEDFEHKQAIMKHYAAENKGLGNLEERQDVVHEDPSLIP
KKMDKCENCAIHDGFAKGFNETMEHAGPQIEKYLGNNTDYKMFVIGHSLGAAQAQLFATQ
FKLLGYDPYMINFGQPRLGNPEFAAYINQLWFNDTGLTVNEARRFYRVTHWNDIVVGVPD
WLNYTHSIGEVFIDEESVYPKLDKVVVCEGGENPLCHRGTFNLWQRINFLQNHLAYIFYI
GLCAFNIGRRDVLNMPTYHGNFSYRNSTDPNYDYNTKAPTRITYPN
>YALI0E08492g LIP4
MAGFNFTFGQVISYLASMLYGQVDATSSSTRIQATQDLYDFTAKFSRLSNIAYCINAPFT
PLRTDFTCGESCRYFPDLQLDSVFGGNFSSASTTGYIAYDHKKKEKYIVFRGTFSIPDII
TDIQFQTAPWLTSLPTHLIPTKEDFEHKQAILKHYAAENKGLSNLEERQDVVHEDPSLVP
KKMDKCENCQIHDGFAKGFNETIEHAGPQIEKFLGNNTDYKMFVVGHSLGAAQAQLFATQ
FKLLGFDPYMINFGQPRLGNPEFAAYINQLWFNDTGLVVNDARRFYRVTHWNDIVVGVPD
WLNYTHSIGEVFIDEESVYPKLDKVVVCEGGENPLCHRGTFNLWSRINFLQNHLAYIFYI

GLCAFNIGRRDVLNMPQYQGNFSYQHNIDPNYNYDTKVPTRISKSN
>YAYA0S2-02982g LIP5
MKFSATLLLCAGTFASAAAIGPLLRRDNQTQIINGFEITDFFNDGEEFLYIKDLTNSSSL
GDNNDLNSRDILGAWESSDTAAVSPADFDYLERQAKLANIAYCGGSSLLTIPFSCAYQCK
EFPNMTLVTTWGDSDTVSPLVAGYLSIDHNAKEIVVGFRGSHTLKDWIVDLIVIREAVDT
AYPGCDDCRVHMGFYDSYQATLAEFETDLKTLAAENPGYRLNVVGHSLGGAVALLAATEF
KRQGYNTYLTTFGQPVVGNTAFAKHVNNHWFGSETPNTLEGNSSRQYYRVTHDSDIVPRV
PFWPGYTPNAGEVYIDVPQIGPVVSDLQFCDGEINYQCVYGNSLLSLISLTAHHNYFVYI
GGDC
>YAYA0S4-04016g LIP5p [pseudogene]
MKF*TTILLCTGTLVASAAIDPVVALDNQTEIFHDYEILDNQTEIINGFEITDFFNEEGE
EFIFIKDLTNSSLLEDNFEDILSSRKLGAWKSSTTGAVSQTSFDYLQKQAKLANIAYCGG
ASLMSAPFICAYQCKEFPNMTLASTWGNSDNHSPLVAGYLAVDDSEKSIVVGFRGSHTLK
DWILDLLVIRRAVDHAYPGCSGCRVHQGFYSAYVATLAYFDADLKKLVAENPGYRVNVVG
HSLGGAVAVLMAATFQNRGYDTYLATFGQPVVGNTAFANYLDRLWFGSENPKTLGDDPSR
RYYRVTHKSDIVPRVPFWPGYTPSSGEVYINVPQTDPSVSDLKTCDGQLNYKCIYGNSLL
SLISMTAHSNYFVQMGGDC
>YAAL0S02-16094g LIP5
MKLSTALLSLCLLGGVSAAPTLGDATKISVVNGFQVAEFIGEDGTEKIVFTDLLNSTWAA
PDLSGLTRSLLGYPEDSGDAEDSDLEERAPVAKALTQETYNYLEKQVKLANIAYCGGTSQ
LISPFVCAYQCKEFPTVELVTTWDTDGLNVSPAVAGYLAIDHESKEFILGFRGSKTLKDW
LVNLNTIRVPVNKKYAPCKGCEVHLGFYNAYKATLGIFESTLTYLRSKHPEYRLNVVGHS
LGGAVALLVATDFKQRGYDTQLTTFGQPIVGNTKFANHIDNLWFGTQAESSTGQFHRVTH
RNDIVPLVPFWLGYSQTAGEIFIGAPNLAPPLDSLRVCEGRNSRECLNGNSILGLLELSA
HTEYFVALGGDC
>YAGA0E32462g LIP5a
MKFSATFLLCTVSLVAAATIAPLAPRDNQTKVINGFQISDLVNDDGDEFLLFKDLTNSSW
PGDTEEDNLYSGVIKAWESTEVAAVSQTSYSYLDKQAKLANIAYCGGSSLLTSPFTCAYQ
CKEFPNMKLVTTWGDSATLSALVAGYLSIDHTDKEIVVGFRGSHTLKDWIVDLMVLRKAV
DDSYPGCDDCRVHHGFYNAYKATLPKFDNDLKKLVAENPGYRVNVVGHSLGGSVALLAAT
EFKNRGYDTYLTTFGQPVTGNTAFANYVDNLWFGSETPKTLSGDSSRRYYRVTHKSDVVP
RVPFWAGYTQNAGEVYIGVAQIDPPVSSLHYCEGEVNHQCVYGNSLLSLISLTAHHNYFV
YIGGDC
>YAGA0F11166g LIP5b
MKLLTTTLIITLASAATLPLISTNSTTIINGFQISNLINDDGDELLLFEDLTKDNVFANS
SLKSDSTLSIRQSSTSPISQTTYNHLVRQSKLANIAYCSGPPSLIAPFTCSYQCKEFPNM
NLVSTWGEDPLSPSVSVAGYLSVDHTEKTIVVGFRGSQTFKNWIVNFMVLRNPVENSYQG
CEGCTVHQGFYNAYKDTKKEYDTELIKLVEENPGYIVNVVGHSLGGSVALLAATDFKNRG
YKTSLTTFGQPVTGNTAFANYIDNLWLTNDTKNTTRSYYRVTHKGDVVPRVPFWHGYSPT
AGEVYIGAAELNPPVSSLLDCEGQENEQCIQGNSLLSLVNLTAHNLYFVYLGGECEVTSL
>YALI0E02640g LIP5
MKFSATLLFCASTLIAAATIAPLAPRGNQTKIINGFQVTDLVNDAGDELLLFKDLTNSSW
PGDTSTSTNGVTLYSGAIKAWTSSDVAAVSQTSYNYLEKQAKLANIAYCGGSSLLTAPFT
CAYQCKEFPNMKLVSTWGDSRTLSALVAGYLSIDHTDKEIVVGFRGSHTLKDWIVDLMVL
RKAVDDSYPGCDNCRVHHGFYSAYKATLARFDNDLKKLVAENPGYRVSVVGHSLGGAVAL
LAATDFKNRGYDTYLTTFGQPVVGNTGFANYVDDLWFGSETPNTLSGDSSRRYYRVTHKS
DVVPRVPFWPGYTPNAGEVYISVAEINPPVSALRYCDGEVNDQCLHGNSLLSLINLTAHH
NYFIYIGGDC
>YAAL0S04-02696g LIP7
MVQIGKFTEWLSVTLWGAAATTSSTATSSITQNTYDFVRTFSHLSNVAYCVKAPIKSLDD
NFQCGNACKNFPNMELVTTFGGDFFQTSITGFLALDHVKKEKYVVYRGTFSIADVITDLQ
FQQSGFLVDAPALNSLKANDTSESAKIDCKDCKIHDGFKKANTETMTNIGDDLKKHLDSY
PDYKLYVTGHSLGAAQALLSAISIKLQGYDPTLINFGQPRVGNAAFANYVDRLFFGEDAG
LSVTSDRKLYRLTHWNDVFVGLPNWDGYQHNVGEVFIDWRFTNPPLQYVKSCEGGENPKC
YRKDFNLLAQINLLQNHLAYIDYIGYCTLNIGRRAQMNLPRYTGPNTYAHKTEDDVELAL
Y
>YAGA0D10484g LIP7
MVNFGARVKDFFSVLLFGAASTTSSTKTALVSQGFYDAALDFSHLSNIAYCVNAPITPLK
DDFSCGQSCVHFPDIELVHTFGGDFFSTSITGYLALDHVKKEKYVVFRGTFSIADAITDI
QFQQSSFLVNVPALNTFTANDTSAEAQIDCKHCKIHDGFSKAFTETLHNIGPQLKQHLDS
YPDYQLYVTGHSLGAAMALLAGTSIKLKGYDPILINYGQPRVGNKAFADYISTLWFGKGD
GLEINKDRRLYRMTHWNDVFVGLPNWDGYTHSNGEVYIKGKYVNPPLKDLMSCAGGENSK
CYRSTFNLLAQINLLQNHLCYIDYIGFCALNVGRRQLNDMPHYTGPYTYGHKTEEDFVAE
GLELSN

>YALI0D19184g LIP7
MVSFGARIKDFFSVLLFGAASTSSSTKTALVSQGFYDAALDFSHLSNIAYCVNAPITPLK
SDFSCGQSCVHFPDIELVHIFGGDFFSTSITGYLALDHVKKEKYVVFRGTFSIADAITDI
QFQQSSFLVNVPALNTFIANDTAPEAQIDCKQCKIHDGFSKAFTETWHNIGDLLEQHLDS
YPDYQLYVTGHSLGAAMALLGATSIKLRGYDPILINYGQPRVGNKAFADYISALWFGNGD
GLEINQQRRLYRMTHWNDVFVGLPNWDGYTHSNGEVYIKGKYVNPPLKDVFSCAGGENSK
CYRSEFNLLAQINLLQNHLCYIDYIGFCALNVGRRELNDLPHYNGPYKYGHKTEEQFIAE
GLELSN
>YAGA0F22892g LIP8
MVSLSARIKDFFSVLLLGAAEITPSTQTAGVSQGFYDFARDFAHLSNVAYCVDAPITPLK
ADFTCGNACKHFPDIELVKTFGGDFFDTSITGFLAVDHVKKEKYVVFRGTFSLADAITDM
QFQQSPFLVDVPAMNTFSANDTAAEAQTQCEGCKIHDGFSKAFTETWGEIGEDLHKHLDS
NPDYQLFVTGHSLGAAMALLGATSIKLKGYDPILINYGQPRVGNKPFSEFINKLWFGDDN
GLEIKPERRLYRMTHWNDIFVGLPNWEGYTHSNGEVYINNRFINPPLKDVISCAGGENSQ
CYRSSFNLLSQINLLQNHLAYIDYIGYCALNIGRRELADQPHYKGNYFYAHRTEEDFKKL
GLELSTRTKQ
>YALI0B09361g LIP8
MVSLSARIKDFFSVLLLGAATITPSTQTAGVSQGFYDFARDFAHLSNIAYCVNAPITPLN
PDFTCGNSCKHFPEIELVKTFGGNFFKTSITGYLAVDHVKKEKYVVFRGTFSLADAITDM
QFQLSPFLVDVPALNTFSANDTTAEAQTHCEGCKIHDGFSKAFTETWGNIGEDLQKHLDA
NPDYQLYVTGHSLGAAMALLGATSIKLKGYDPILINYGQPRVGNKPFAEFINKLWFGEGN
GLEITPERKLYRMTHWNDIFVGLPNWEGYTHSNGEVYINNRFINPPLKDVISCAGGENSK
CYRSSFSLLSQINLLQNHLAYIDYIGYCALNIGRRELADQEHYTGPYYYGHRSEEDFKKL
GLELSTPQVEN
>OLHI0E00452g LIP9
MLIWLPLVLCVSQKLLAELTRYMRFAEVAGCVGSGSRGQKLPGITRPFQCPLCDLPVFGS
AELLGTFSSAYFEKQPTGYIAVDTYHGELLVVFRGTTSFGDKIADFSIFKKRVCEVEALQ
EHLDMIDDNAQIFGGVVDVFNGGFEPLIWQIKRALMKYPGYRLVVLGHSLGGAIATLCAV
TLKLRGFNPYLITFAEHLTGNWEWAQTVNWLFPGKTSYRVTRVGDIVPRLPPTELGYYPR
GSEYYISSYEMPLHHEELFYCDESNHCSQRAPLWSYLLNYQETQKSHFNYFFELRKCTA
>YAYA0S2-34200g LIP9p [pseudogene]
MRLATIASFVGLVTASPIGWIPPALLNPFGPRKPDGTVVNATLATVNEMEHYWKFCSVSY
CVGIGNNNQLYSQIKTPFVCNNILCADEEFSETELLYSFYGVNEHQTANGYIAVDHKAKQ
LVLVFRGTQDEADTAADLNTWQVSNVDFSGITNASDTNAQSSCPGCSLHAGFVGIFNYSF
KQINGRLNLYKATYPDYKLVVTGHSLGGAIALLYGVSLKINGRDPLVVTFGQPRVGNAAF
ATYVDSLFLPTAGDQLTTSPSRNMYRVTRYEDPVTQVPFWAGYTQQSGEVYINQFSVPTN
AENVMLCQGQNNGFCANGIPWYQYANLDSDKQLHSSYFFRSPSCGGSQSYVPYGSNNTEP
TSAITIPPGSLQNQLSLPYDIPN
>YAAL0S08-03510g LIP9
MRIAIFASLLALVQASPIGIVPPVFLPLLGGKGADGTIAKAPVSIVDEMEHYWKFCSVSY
CVGMGKNDQLYSQVRQPFVCNNVLCADQEFSQTEVLYSFYGINEHQTANGYIAVDHKRKQ
LVLVFRGTQSEADSAADLNTWQVPNVNFDGLKNTTGTNAESDCQGCSIHAGFVGIFNNSF
KAIDSRLNLYKAQYPDYKLVVTGHSLGGAVALLYGISLRVNGRDPLVVTFGQPRVGNEAF
ANYVDSLFFPSLNDMLSYSPYRKMYRVTRYEDPVTQVPFWDGYTQQSGEVFINQFKVPTD
PENVLFCQGQNNRNCANGIPWYQYANIASDKSVHSSYFFRSPSCSNAQAYTPYGGNTTAA
PGAVSLDPSELKTRLNLPYDF
>YAGA0E00738g LIP9
MKLAAIASFVGLVAASPIGLLPPALLNPFGPKKPDGTVAPAPLDVVTEMEHYWKFCSVSY
CVGMGKNNQLYSQVKTPFVCNNILCADKEFSETELLYSFYGINEHQTANGYLAADHKRKQ
LVLVFRGTQSEADSAADLNTWQVSNVDFDGLKNSTSTNAESECQGCSIHAGFVGIFNNSF
KQIDSRLNLYKGMYPDYKLVVTGHSLGGAVALLYGVSLKINGRDPLVVTFGQPRVGNAAF
ASYVDSLFFPTASDQLSSSPYRKMYRVTRYEDPVTQVPFWDGYTQQSGEVFINQFKVPTD
PKNVVFCQGQNNGYCANGIPWYQYANIDSDKQVHSSYFFRSPGCAGTQSFTPYGGNTSEP
SAVSLTPGDLKARLNLPYDTNY
>YALI0E34507g LIP9
MYNGIAWEGYIYILGPFLKTRQHQKPQDTIMRLATIASFVGLVTASPIGLLPPALLNPFG
PKKPDGTVATAPLDVVHEMEHYWKYCSVSYCVGMGKNNQLYSQVKSPFVCNNILCADQEF
SQTELLYSFYGINEHQTANGYLAADHKRKQLILVFRGTQSEADSAADLNTWQVSNVDFDG
LKNSTDTNAESDCHGCSIHAGFVGIFNNSFKQIDSRLNLYKSMYPDYKLVVTGHSLGGAV
ALLYGVSLRINGRDPLVVTFGQPRVGNAAFASYVDSLFFPTAGDQLSSSPYRKMYRVTRY
EDPVTQVPFWDGYTQQSGEVFINQFNVPTKPENVVFCQGQNNGFCANGIPWYQYANIDSD
KQVHSSYFFRSPGCGGSQSFTPYGGNQTEPSAVSIPPGDLKARLNLPYDTNY
