# Supplementary material for: Comprehensive Analysis of a Yeast Lipase Family in the Yarrowia Clade
Source: PLoS One. 2015 Nov 18;10(11):e0143096. doi: 10.1371/journal.pone.0143096 (PMC4651352; doi:10.1371/journal.pone.0143096)
Supplement: S3 Table — (DOCX) [file pone.0143096.s007.docx]

**Additional Table S3:** Primers used for lipase cloning

| **Enzyme** | **PrePro region** | **Primer sequence** |
| --- | --- | --- |
| YlLip2a | PrePro YlLip2 | GACGAGTGTACACCTCCACGGAGACTTCCCAGATCAG |
|  |  | CCAAGGCATTTGCGGCATCTAACCATCCTAGGATGTAA |
| YlLip2a | PrePro YxLip2 | CCGAAGGATCCCACAATGAAGTTTCTAAGTCTCGCTG |
|  |  | ACCCTAGGTACCTTAGATGCCGCAAATGCCTT |
| YlLip2b | PrePro YlLip2 | AACGAGTGTACACCTCTACTGAAACCGCTGAAGTCAGTG |
|  |  | GTGCTCATGGGCATCTGTGGTATCTAACTTTCCTAGGATTGAT |
| YlLip2b | PrePro YxLip2 | CCGAAGGATCCCACAATGAAGCTCTTCACACTCGCTCTTG |
|  |  | ACCCTAGGTACCTTAGATACCACAGATGCCCATGAGCACAAAG |
| YlLip2c | PrePro YlLip2 | AACGAGTGTACACCTCTACTGAAACCGCTGAAATCAGTG |
|  |  | GTTCTCATGGGCATCTGTGGTATCTAACTTTCCTAGGATTGAT |
| YlLip2c | PrePro YxLip2 | CCGAAGGATCCCACAATGAAGCTCTTCACGCTCGCTCTTG |
|  |  | ACCCTAGGTACCTTAGATACCACAGATGCCCATGAGAACAAAG |
| YlLip2d | PrePro YlLip2 | TCCGCCTGTACAAATCCACAGAAACCTATCACATTGATCAG |
|  |  | GTTCTTCAGGGTATCTGCGGCTTGTAGTAGGCCTAGGAGATCT |
| YlLip2d | PrePro YxLip2 | CCGAAGGATCCCACAATGAATTTCTTCAAGATCTTTCTACTTTCAGC |
|  |  | ACCCTAGGTACCTTACAAGCCGCAGATACCC |
| YlLip2e | PrePro YlLip2 | AGCGAGTGTACACATCCACAGAGACTGATCCTGTGACC |
|  |  | GTAAGTGCAACGCTTTCAACACTTTTAGTGATTAGTTAACCTAGGATGAAT |
| YlLip2e | PrePro YxLip2 | CCGAAGGATCCCACAATGAAATTGACTAACCTTCTTGCTG |
|  |  | ACCCTAGGTACCTTAATCACTAAAAGTGTTGAAAGCGTTG |
| YgLip2 | PrePro YlLip2 | CCAGAAGCGAGTGTACACCTCTACCGAGACTTCC |
|  |  | GATAAATACCCTAGGTTAGATACCACAGACACCCTC |
| YgLip2 | PrePro YxLip2 | CCGAAGGATCCCACAATGAAGCTTTCTACCATCCTTCTTACAGC |
|  |  | ACCCTAGGTACCTTAGATACCACAGACACCCTCG |
| YyLip2 | PrePro YlLip2 | GAAGAGAGTGTACACTTCTACCGTGACCACTCCC |
|  |  | GAAGAAGTGATACCTAGGTTAGATACCACAGATACC |
| YyLip2 | PrePro YxLip2 | CCGAAGGATCCCACAATGAAGATCCAAAACATCCTCCT |
|  |  | ACCCTAGGTACCTTAGATACCACAGATACCCTGG |
| YaLip2 | PrePro YlLip2 | CAGGAGCGAATGTACACCTCTACCGAGACC |
|  |  | GAAAGAGAAGCCTAGGGTAACTAAATACCACAAACACCTC |
| YaLip2 | PrePro YxLip2 | CCGAAGGATCCCACAATGAAGCTTTCCACCCTCGTCCTCAC |
|  |  | ACCCTAGGTACCTTAAATACCACAAACACCTTCAGTGACAAAGTACTGGAG |
| YhLip2 | PrePro YlLip2 | AAGCTCGAGTGTACATTAGTGTAGAGACTGCTCAAG |
|  |  | GGCACTATCCTAGGATGTCTTACAAAGCTAGGCAAT |
| YhLip2 | PrePro YxLip2 | ACCCTAGGTACCTTACAAAGCTAGGCAATTAGCCT |
|  |  | CCGAAGGATCCCACAATGAAGTTCCTCACTGTTC |
| YlLip2d F163L |  | CACTCTCTGGGAGGAGCAACTGCCCTTTTG |
|  |  | TCCTCCCAGAGAGTGCCCTGCAACAACCAT |
| YlLip2a D97A |  | ATCACTGCTCTTCGAATTAAGCAGGCTCCT |
|  |  | TCGAAGAGCAGTGATGACATCTTCCAAGGA |
| YlLip2a V232F |  | GATATTTTCCCTCAGATTCCTTTCTGGGAT |
|  |  | CTGAGGGAAAATATCTCCTCGGTGAGTAAC |
| YlLip2a V232S |  | GATATTTCCCCTCAGATTCCTTTCTGGGAT |
|  |  | CTGAGGGGAAATATCTCCTCGGTGAGTAAC |
